# The Human PDZome 2.0: Characterization of a New Resource to Test for PDZ Interactions by Yeast Two-Hybrid

**DOI:** 10.3390/membranes13080737

**Published:** 2023-08-17

**Authors:** Monica Castro-Cruz, Frédérique Lembo, Jean-Paul Borg, Gilles Travé, Renaud Vincentelli, Pascale Zimmermann

**Affiliations:** 1 Department of Human Genetics, KU Leuven, 3000 Leuven, Belgium; castrocruzm@gmail.com; 2 Équipe Labellisée Ligue 2018, Centre de Recherche en Cancérologie de Marseille (CRCM), Aix-Marseille Université, 13009 Marseille, France; fredlembo@yahoo.fr; 3 Marseille Proteomics Platform, CRCM, Institute Paoli-Calmettes, Aix-Marseille Université, Inserm, CNRS, 13009 Marseille, France; jean-paul.borg@inserm.fr; 4 Équipe Labellisée Ligue 2015, Department of Integrated Structural Biology, Institut de Génétique et de Biologie Moléculaire et Cellulaire (IGBMC), INSERM U1258/CNRS UMR 7104/Université de Strasbourg, 67404 Illkirch, France; travegi@igbmc.fr; 5 Architecture et Fonction des Macromolécules Biologiques (AFMB), Unité Mixte de Recherche (UMR) 7257, Centre National de la Recherche Scientifique (CNRS), Aix-Marseille Université, 13009 Marseille, France; renaud.vincentelli@univ-amu.fr

**Keywords:** PDZ/PDZome, protein–protein interaction, HPV16-E6, yeast two-hybrid

## Abstract

PSD95-disc large-zonula occludens (PDZ) domains are globular modules of 80–90 amino acids that co-evolved with multicellularity. They commonly bind to carboxy-terminal sequences of a plethora of membrane-associated proteins and influence their trafficking and signaling. We previously built a PDZ resource (PDZome) allowing us to unveil human PDZ interactions by Yeast two-hybrid. Yet, this resource is incomplete according to the current knowledge on the human PDZ proteome. Here we built the PDZome 2.0 library for Yeast two-hybrid, based on a PDZ library manually curated from online resources. The PDZome2.0 contains 305 individual clones (266 PDZ domains in isolation and 39 tandems), for which all boundaries were designed based on available PDZ structures. Using as bait the E6 oncoprotein from HPV16, a known promiscuous PDZ interactor, we show that PDZome 2.0 outperforms the previous resource.

## 1. Introduction

PDZ scaffold proteins are involved in a wide range of cellular processes, including the establishment and maintenance of polarity, protein trafficking, signaling and the coordination of synaptic events [1,2,3]. They contain one or more PDZ domains, an abundant and promiscuous protein interaction module. PDZ domains were first identified in the proteins PSD-95 (postsynaptic density-95), Dlg-1 (disc large-1), and ZO-1 (zona occludens-1) [4,5,6,7,8]. PDZ domains generally recognize short linear motifs of ±4 amino acids (PDZ binding motifs or PBM) located at the C-terminal region of receptors, co-receptors, or membrane adhesion molecules [9]. Some PDZ domains can interact with internal binding motifs, lipids and other PDZ domains [10,11,12]. PDZ interactions can be tuned in various ways. Changes in salt content and pH [13], autoinhibition [14], allosteric regulation [15] and phosphorylation [16] are some of the features that modulate PDZ interactions (for reviews see [17,18,19]).

PDZ domains are composed of 80–90 amino acid residues which fold in six β-strands (A–F) and two α-helices (A–B), forming a partially opened antiparallel B barrel structure [1,20]. The PBM binds in a groove formed by the α-helix B and the β-sheet B [20]. The PDZ binding groove is connected by a loop that often contains the GLGF motif. The GLGF motif, also described as R/K-X-X-X-G-φ-G-motif where X is any and φ is an hydrophobic residue, can vary significantly and contributes to the affinity of the interactions with the PBM [20,21]. Structural and functional studies suggest that PDZ domains prefer specific residues in a PBM. One can currently identify three main PBM classes and up to 16 specificity sub-classes [21]. Yet, approaches like, e.g., phage display suggest that PBM specificities go beyond such classification [22,23].

PDZ domains are rare in non-metazoans. For example, bacteria and yeast display no more than 2 and 4 PDZ-domain containing proteins, respectively [24,25]. In contrast, PDZ proteins are abundant in metazoans, suggesting they co-evolved with multicellularity [25]. Characterization of the functional relevance of PDZ proteins in cells and organisms amply highlighted their important organizational role at membranes, being, i.e., required for proper functioning of cell junctions [5,7,8] or intracellular trafficking [2,3]. Not surprisingly, in retrospect, PDZ proteins can often also directly interact with lipids and structure-function studies established the functional importance of these interactions [12,26,27,28,29]. Before 2012, several studies based on sequence analysis using SMART (www.smart.embl-heidelberg.de), Interpro (https://www.ebi.ac.uk/interpro/), and PFAM (https://pfam.xfam.org/) suggested that the number of PDZ domains in the human proteome ranges from 234 to 450 [30,31,32]. Based on these strictly in silico studies, a first collection of human PDZ domains was built (PDZome) to test for PDZ interactions by Yeast-two-hybrid (Y2H) [30]. This resource contains 246 PDZ domains. Yet according to a more refined study including a 3D-structure based approach and careful manual annotation, this resource contains many PDZ domains truncated at their N- and C-termini by 5 to 16 amino-acids [33]. Such truncation might compromise proper folding and binding activities [28,33,34,35]. In the current more refined study, we identified 266 PDZ domains embedded in 150 proteins (omitting spliced forms) in the human proteome.

Of note, it became clear that some PDZ domains occurring in tandem (separated by a short conserved linker region) can function as supramodules [36,37]. The binding properties of these supramodules are different from those of PDZ domains taken in isolation. Generally, PDZ tandems display higher affinity for their target and in some cases the tandem might be necessary for the proper folding of individual domains [37,38].

Because the original PDZome resource [30] misses some PDZ domains and does not contain tandems, and also because of the presence of suboptimal boundaries, we prepared a new resource that we called PDZome 2.0 [39]. The PDZome 2.0, is more comprehensive including the 266 manually annotated sequences of single PDZ domains [33]. Additionally, it contains 39 PDZ domains in tandem. To test the performance of PDZome 2.0, we used the E6 oncoprotein present in human papilloma virus-16 (HPV16). The PDZome 2.0 detected a total of 54 E6–PDZ interactions. Twenty-nine were common with the 36 previously identified by the PDZome, and 25 were newly identified. We therefore propose the PDZome 2.0 as a more performant resource to comprehensively map human PDZ interactions by Yeast-two-hybrid approach.

## 2. Materials and Methods

### 2.1. Sub-Cloning of Prey and Baits

First, prey genes were synthesized and cloned into the pHTP0 vector following established protocols (NZYTech, Ltd., Lisbon, Portugal). Subsequently, the genes were cloned into the pDONR Zeo (or pDONOR201) entry vector (Addgene, Cambridge, MA, USA) using Gateway^®^ BP reactions [39]. All the entry clones were subcloned into the Yeast Two-Hybrid (Y2H) expression vector pACT2-AD using Gateway^®^ LR reactions (Thermo Fisher, Waltham, MA, USA). After sequence validation, all pACT2-AD clones were transformed into the haploid Y187 yeast strain (MATα, ura3-52, his3-200, ade2-101, trp1-901, leu2-3, 112, gal4Δ, met-, gal80Δ, MEL1, URA3::GAL1UAS-GAL1TATA-lacZ).

The two baits used here correspond to a fragment of the HVP16 E6 oncoprotein wildtype (MSCCRSSRTRRETQL), and the same fragment without the PDZ binding motif or ΔTQL (MSCCRSSRTRRE). The E6 fragments were subcloned into the pGBT9-BD vector for expression in yeast, as reported previously [30]. After sequence validation, E6 constructs were transformed into the haploid AH109 yeast strain (MATa, trp1-901, leu2-3, 112, ura3-52, his3-200, gal4Δ, gal80Δ, LYS2::GAL1UAS-GAL1TATA-HIS3, GAL2UAS-GAL2TATA-ADE2, URA3::MEL1UAS-MEL1 TATA-lacZ).

### 2.2. Yeast Two-Hybrid Assays

PDZ interactions were tested and screened by Y2H assay [40]. Briefly, the Y2H was performed through mating of the two yeast strains Y187 (α) and AH109 (a). The yeasts were grown together (α + a) in liquid Yeast extract-Peptone-Dextrose (YPD) supplemented with 10% PEG for 5–6 h at 30 °C under gentle agitation (140 rpm). After one wash in sterile water, the yeasts were spotted on solid medium. To test the mating efficiency, the yeasts were spotted on a solid permissive medium SC Agar -L-W. To test for interactions, the yeasts were spotted on a solid selective medium SC Agar -L-W-H. All SC-Agar plates were incubated for at least 72 h and up to 1 week at 30 °C or 2 weeks at room temperature. Images from the solid selective medium plates were captured and analyzed. Random positive clones were verified using PCR amplification and automated sequencing with the GAL-AD primer (Eurofins GATC).

## 3. Results

### 3.1. Construction of the Human PDZ Resource for Yeast Two-Hybrid Assays

**Figure 1:**
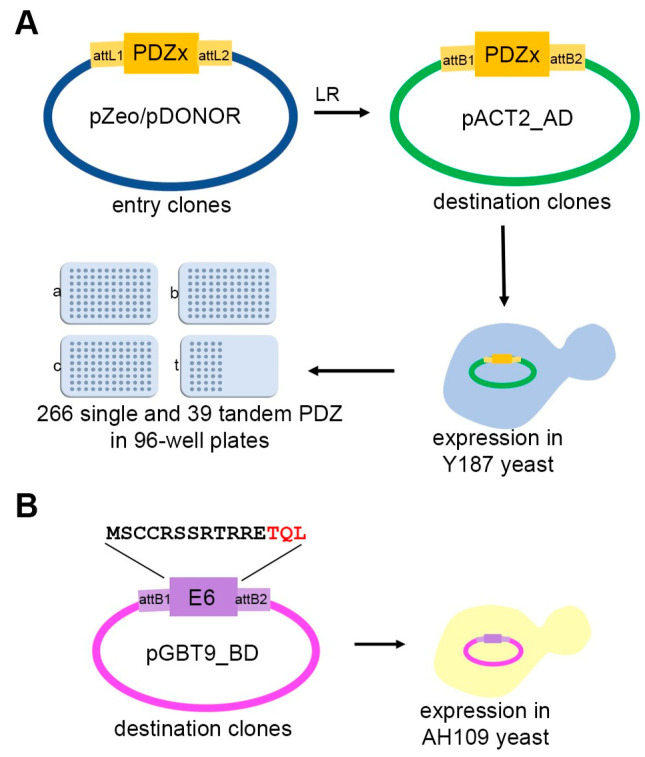
Construction of the PDZome 2.0 for Yeast two-hybrid screens. The PDZome 2.0 was built using the Gateway^®^ cloning system. (**A**) The entry clones corresponding to the open reading frames (ORF) of the 266 PDZ domains and 39 PDZ domains in tandem were subcloned from pZeo or pDONOR entry vectors. The ORFs were then introduced into the pACT2-AD vector using Gateway^®^ LR clonase. After validation by sequencing, pACT2-AD clones were transformed into the Y187 (type α) yeast strain. Ready-for-mating yeast containing the PDZome fused to the Gal4 activation domain were arranged in 4 plates of 96 wells (a, b, c correspond to single PDZ domains, whereas t corresponds to tandems). (**B**) Two peptides corresponding to the C-terminal part of the E6 protein from HPV16 were used as baits. The wild type (MSCCRSSRTRRETQL) and the ΔTQL (or ΔPBM) were subcloned in the pGBT9-BD vector as described previously [30] and transformed into the AH109 (type a) yeast strain.

To build the human PDZome 2.0 resource allowing to test for PDZ interactions by yeast two-hybrid screenings (Y2H), the 266 known human PDZ domain sequences, bearing optimized boundaries based on available structural data [33], were introduced in the prey vector using a Gateway^®^ approach (Figure 1A). We also included 39 PDZ tandems (Tables S1 and S2). The PDZ tandems were designed using the online UniProt resource (https://www.uniprot.org/, accessed on 5 May 2017). First, all PDZ proteins with more than one PDZ domain were included in the list (multi-PDZ proteins). Then, within these multi-PDZ proteins, those in which 2 PDZ domains were connected by a linker region of up to 36 amino acid residues acids were included. The final list of 39 tandems, belonging to 28 PDZ proteins, represented around 20% of the human PDZ proteome (Table S2).

All recombinant clones present in the prey pACT2-AD vector were transformed into the haploid Y187 (α) yeast strain. The final collection of individual clones was arrayed in four 96-well plates (Figure 1A).

### 3.2. The PDZome 2.0 for Y2H Screenings Is Validated Using the HPV16 E6 Oncoprotein

To characterize the performance of the PDZome 2.0, we used a fragment of the HPV16 E6 oncoprotein as bait in the Y2H screenings. The HVP16 E6 oncoprotein is involved in the development of human cervical cancer by exploiting its class I PBM, which has been previously described to bind at least 29 PDZ scaffold proteins [30,41,42,43].

Two E6 constructs were used to validate the new resource. These were the wild-type E6 (MSCCRSSRTRRETQL) and the mutant E6 ΔPBM, in which the PBM is disrupted by removing the last 3 amino acids (MSCCRSSRTRRE) (Figure 1B). Bait constructs were subcloned in the pGBT9-BD expression vector and fusion proteins were expressed in the AH109 yeast strain for Y2H (Figure 1B).

**Figure 2:**
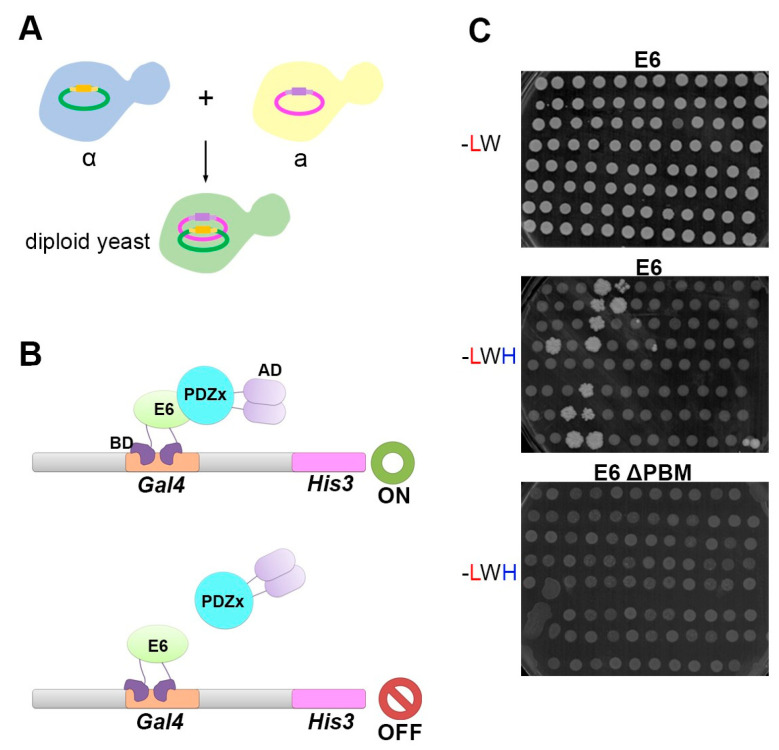
Y2H mating and selection process. (**A**) Scheme illustrating the mating of the two yeast strains. The ‘a’ type yeasts hosting the E6-pGBT9-BD baits and the ‘α’ type yeasts hosting the PDZome 2.0-pACT2-AD were allowed to mate. Diploid yeasts containing both the PDZ and the E6 constructs were selected in synthetic agar medium. (**B**) Scheme illustrating the detection of protein interaction by Y2H. In case the E6-bait coupled to the Gal4 binding domain (BD) interacts with the given PDZ-prey coupled to the Gal4 activation domain (AD), the HIS3 reporter gene is expressed, allowing the growth of the diploid yeasts in a synthetic medium without histidine. In case there is no interaction between the bait and prey, the AD is not recruited and the HIS3 reporter gene is not expressed. (**C**) Photographs exemplifying the growth of diploid yeasts containing both the PDZ and the E6 constructs. Diploid yeasts are selected in permissive culture medium without leucine and tryptophan (-LW). White dense colonies in the -LW medium suggest effective mating (upper panel). Simultaneously, the phenotypic test for interactions was performed in selective culture medium without leucine, tryptophan, and histidine (-LWH). White and dense colonies in the -LWH medium correspond to interaction pairs (middle panel). Disruption of the PBM effectively impairs the appearance of white dense colonies in the -LWH medium, confirming a PBM-mediated mode of interaction (lower panel).

Y2H screens were carried out by mating the two recombinant yeast strains Y187 (α) and AH109 (a), allowing the formation of diploid yeasts expressing the prey and the bait constructs (Figure 2A). According to Y2H principles, in case the E6 bait interacts with a given PDZ prey, a complex is formed and the activating domain (AD) is recruited near the reporter gene, where it can stimulate its expression (Figure 2B,C). To control mating efficiency, we cultured our mated yeasts in SC-Agar medium lacking leucine and tryptophan (-LW). The growth of dense white colonies indicated efficient mating (Figure 2C upper panel). Simultaneously, to test for PDZ interactions, the mated yeasts were grown in SC-Agar medium lacking leucine, tryptophan, and histidine (-LWH). The growth of dense white colonies in the medium -LWH was indicative of E6–PDZ interactions (Figure 2C middle panel). As expected, when the E6 PBM was disrupted (E6 ΔTQL), yeasts failed to grow in the -LWH medium (Figure 2C lower panel), indicating that the PBM is essential.

**Figure 3:**
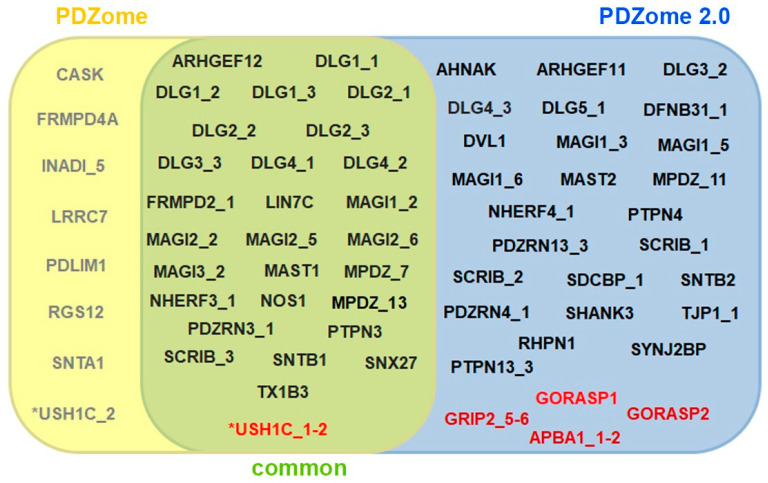
Mapping of E6–PDZ interactions using PDZome 2.0 compared to the previous resource. Venn diagram representing the positive interactions identified by Y2H screens using the first PDZome (yellow) and PDZome 2.0 (blue). Common interactions detected using both resources are shown in the intersection region (green). Interactions revealed using PDZ tandems are highlighted in red. * Note that the USH1C interaction was detected using the PDZ 2 domain taken in isolation, as present in the first PDZome, and using the tandem (USH1C_1-2) from PDZome 2.0.

We identified 53 PDZ domains interacting with the PBM of the E6 protein from the HPV16. When applicable, these interactions were confirmed with the tandem constructs. In addition, the tandems identified four interactions not detected when PDZ domains were taken in isolation (Figure 3 and Figure S1). Globally, the PDZome 2.0 outperforms the previous PDZome version, the latter identifying solely 36 PDZ domains interacting with E6 [30] (Figure 3). Nevertheless, eight interactions observed with the PDZome were not detected with the PDZome 2.0. In total, the PDZome 2.0 identified 43 PDZ proteins and 57 PDZ domains able to interact with the E6 protein of the HPV16. The previous version of the PDZome detected 28 PDZ proteins and 36 PDZ domains.

## 4. Discussion and Conclusions

In this study, we built and tested a new, and today’s most comprehensive, resource to test for human PDZ interactions by Y2H. Compared to the previous version [30], this resource contains 20 additional PDZ domains in isolation (266 instead of 246). Moreover, PDZ domains are flanked by extended boundaries meant to insure proper folding [33]. Finally, PDZome 2.0 contains 39 PDZ domains in tandem. We thereby aimed to provide more reliable materials to test for human PDZ interactions in a comprehensive manner.

When compared to the previous version, the PDZome 2.0 was shown to be outstandingly efficient in the production of the soluble protein in the BL21 (DE3) pLysS *E. coli* bacteria strain (35). These effects were most probably due to their optimized boundaries [33].

Consistently, the PDZome 2.0 revealed 25 interactions that were not detected previously for the viral oncoprotein E6. Among those 25 interactions, 9 were previously detected using the chromatographic holdup approach (HU) [43,44]. Curiously, the PDZome 2.0 failed to detect 7 interactions that were detected with the previous version of the PDZome. The reasons are unclear. One possible reason could be that the extended sequences in the PDZ domains restrain particular interactions or contribute to the autoinhibition of the PDZ domain [14,33]. Alternatively, these interactions might correspond to false positives [45]. Additionally, there are certain discrepancies in the identities/names and/or sequences between the first PDZome and the PDZome 2.0 [30,39,43,44], blurring the distinction of failures due to technical or performance issues. To see some examples, go to the Table S1.

The presence of tandem structures in a protein (i.e., co-folding domains) can enhance the affinity for a particular ligand [36]. Consistently, the PDZ tandem constructs not only validated interactions observed with PDZ in isolation but also revealed additional interactions. Three of these extra interactions were not described previously in papers reporting the HPV16-E6–PDZ interactomes [30,41,43,44,46,47,48,49]. Obviously, the PDZome 2.0 might still be prone to a false negative. It is always recommended to verify interactomes using complementary biochemical or biophysical methods such as HU [43,44], phage display [50] or pull-down and mass spectrometry [51]. Then, to understand the function of individual PDZ proteins and their interactome by cell biological, developmental or physio-pathological approaches [26,29,52,53,54,55,56,57], it is useful to define point mutants allowing for structure–function analysis. Therefore, surface plasmon resonance, somehow recapitulating the membrane(2D)-cytosol(solution) and allowing to measure interaction kinetics [58], has been proven to be a reliable approach.

In conclusion, PDZome 2.0 for Y2H represents a valuable additional resource to test for PDZ interactions and is certainly an easy going first-line choice when one aims to investigate the PDZ interactome.

## Data Availability

The data presented in this study are available in the results section and in the supplementary material. All source data and additional results are available from the corresponding author upon reasonable request.

## Supplementary Materials

The following supporting information can be downloaded at: https://www.mdpi.com/article/10.3390/membranes13080737/s1, Figure S1: Summary of the Yeast-Two-Hybrid raw data; Table S1: Single PDZ domain constructs used to comprehensively map PDZ interactions; Table S2: Tandem PDZ constructs used as preys to comprehensively map PDZ interactions.
