# Supplementary material for: The Human PDZome 2.0: Characterization of a New Resource to Test for PDZ Interactions by Yeast Two-Hybrid
Source: Membranes (Basel). 2023 Aug 17;13(8):737. doi: 10.3390/membranes13080737 (PMC10456741; doi:10.3390/membranes13080737)
Supplement: Supplementary file 1 [file membranes-13-00737-s001.zip › membranes-2515547-supplementary.pdf]

# Supplementary Material

**Figure S1 Summary of the Yeast-Two-Hybrid raw data.** Summary of the PDZ interactions detected by Yeast-Two-Hybrid using the E6 protein C-terminal region of HPV16 wild type as bait. (**A**) Comparison of the interactions detected using the PDZome and the single PDZ collection of the PDZome 2.0 (as indicated). (**B**) Comparison of the interactions detected using the collection of tandems of the PDZome 2.0 and the correspondent PDZ taken in isolation in PDZome and PDZome 2.0 (as indicated). No interaction detected is indicated in red, positive interaction detected is indicated in blue. Numbers correspond to positive colonies / detected from *n* independent experiments.

**Table S1. Single PDZ domain constructs used to comprehensively map PDZ interactions.**

**Table S2. Tandem PDZ constructs used as preys to comprehensively map PDZ interactions.**

# Figure S1 Summary of the Yeast-Two-Hybrid raw data

**A**

| PDZ domain | PDZome Belotti et al, 2013 | PDZome 2.0 |
|---|---|---|
| AHNAK | | 1 / 3 |
| ARHGEF11 | | 1 / 3 |
| ARHGEF12 | | 2 / 3 |
| CASK | | 0 / 3 |
| DFNB31_1 | | 1 / 3 |
| DLG1_1 | | 2 / 3 |
| DLG1_2 | | 3 / 3 |
| DLG1_3 | | 2 / 3 |
| DLG2_1 | | 2 / 3 |
| DLG2_2 | | 2 / 3 |
| DLG2_3 | | 2 / 3 |
| DLG3_2 | | 2 / 3 |
| DLG3_3 | | 2 / 3 |
| DLG4_1 | | 2 / 3 |
| DLG4_2 | | 3 / 3 |
| DLG4_3 | | 3 / 3 |
| DLG5_1 | | 1 / 3 |
| DVL1 | | 1 / 3 |
| FRMPD2_1 | | 2 / 3 |
| FRMPD4A | | 0 / 3 |
| GORASP1 | | 1 / 3 |
| INADL_5 | | 0 / 3 |
| LIN7C | | 1 / 3 |
| LRRC7 | | 0 / 3 |
| MAGI1_2 | | 2 / 3 |
| MAGI1_3 | | 1 / 3 |
| MAGI1_5 | | 2 / 3 |
| MAGI1_6 | | 1 / 3 |
| MAGI2_2 | | 3 / 3 |
| MAGI2_5 | | 2 / 3 |
| MAGI2_6 | | 1 / 3 |
| MAGI3_2 | | 3 / 3 |
| MAST1 | | 2 / 3 |
| MAST2 | | 3 / 3 |
| MPDZ_11 | | 1 / 3 |
| MPDZ_13 | | 1 / 3 |
| MPDZ_7 | | 1 / 3 |
| NHERF3_1 | | 2 / 3 |
| NHERF4_1 | | 1 / 3 |
| NOS1 | | 1 / 3 |
| PDLIM1 | | 0 / 3 |
| PDZRN3_1 | | 2 / 3 |
| PDZRN4_1 | | 1 / 3 |
| PTPN13_3 | | 1 / 3 |
| PTPN3 | | 3 / 3 |
| PTPN4 | | 3 / 3 |
| RGS12 | | 0 / 3 |
| RHPN1 | | 2 / 3 |
| SCRIB_1 | | 2 / 3 |
| SCRIB_2 | | 3 / 3 |
| SCRIB_3 | | 3 / 3 |
| SDCBP_1 | | 3 / 3 |
| SHANK3 | | 2 / 3 |
| SNTA1 | | 0 / 3 |
| SNTB1 | | 3 / 3 |
| SNTB2 | | 2 / 3 |
| SNX27 | | 1 / 3 |
| SYNJ2BP | | 1 / 3 |
| TJP1_1 | | 1 / 3 |
| TX1B3 | | 1 / 3 |
| USH1C_2 | | 0 / 3 |

**B**

| PDZ domain | PDZome 2.0 tandem PDZ | PDZome 2.0 single PDZ | PDZome Belotti et al, 2013 |
|---|---|---|---|
| APBA1_1-2 | 2 / 2 | | |
| DLG1_1-2 | 1 / 2 | | |
| DLG2_1-2 | 1 / 2 | | |
| DLG3_1-2 | 1 / 2 | | |
| DLG4_1-2 | 2 / 2 | | |
| GRIP2_5-6 | 1 / 2 | | |
| USH1C_1-2 | 1 / 2 | | |
| GORASP2_1-2 | 1 / 2 | | |

**Table S1 Single PDZ domain constructs used to comprehensively map PDZ interactions.**

| Single PDZ name | Protein sequence |
|---|---|
| AHNAK | EKEETTRELLLPNWQGSGSHGLTIAQRDDGVFVQEVTQNSPAARTGVVKEGDQIV GATIYFDNLQSGEVTQLLNTMGHHTVGLKLHRKGDRSPEPGQT |
| AHNAK2 | QEATEVTLKTEVEAGASGYSVTGGGDQGIFVKQVLKDSSAAKLFNLREGDQLLST TVFFENIKYEDALKILQYSEPYKVQFKIRRQ |
| APBA1_1 | EFKDVFIEKQKGEILGVVIVESGWGSILPTVIIANMMHGGPAEKSGKLNIGDQIMSI NGTSLVGLPLSTCQSIIKGLKNQSRVKLNIVR |
| APBA1_2 | PPVTTVLIRRPDLRYQLGFSVQNGIICSLMRGGIAERGGVRVGHRIIEINGQSVVAT PHEKIVHILSNAVGEIHMKTMPA |
| APBA2_1 | NCKELQLEKHKGEILGVVVVESGWGSILPTVILANMMNGGPAARSGKLSIGDQIM SINGTSLVGLPLATCQGIIKGLKNQTQVKLNIVS |
| APBA2_2 | PPVTTVLIKRPDLKYQLGFSVQNGIICSLMRGGIAERGGVRVGHRIIEINGQSVVAT AHEKIVQALSNSVGEIHMKTMPAA |
| APBA3_1 | DNCREVHLEKRRGEGLGVALVESGWGSLLPTAVIANLLHGGPAERSGALSIGDRL TAINGTSLVGLPLAACQAAVRETKSQTSVTLSIVHS |
| APBA3_2 | PVTTAIIHRPHAREQLGFCVEDGIICSLLRGGIAERGGIRVGHRIIEINGQSVVATPH ARIIELLTEAYGEVHIKTMPAATYRLLTGQ |
| ARHGAP21 | GPKTVTLKRTSQGFGFTLRHFIVYPPESAIQFSYKDEENGNRGGKQRNRLEPMDTI FVKQVKEGGPAFEAGLCTGDRIIKVNGESVIGKTYSQVIALIQNSDTTLELSVMPK D |
| ARHGAP23 | QGPRTLLLYKSPQDGFGFTLRHFIVYPPESAVHCSLKEEENGGRGGGPSPRYRLEP MDTIFVKNVKEDGPAHRAGLRTGDRLVKVNGESVIGKTYSQVIALIQNSDDTLEL SIMPKD |
| ARHGEF11 | TGLVQRCVIIQKDQHGFGFTVSGDRIVLVQSVRPGGAAMKAGVKEGDRIIKVNGT MVTNSSHLEVVKLIKSGAYVALTLLGSS |
| ARHGEF12 (LARG) | TGLVQRCVIIQKDDNGFGLTVSGDNPVFVQSVKEDGAAMRAGVQTGDRIIKVNG TLVTHSNHLEVVKLIKSGSYVALTVQGRPPGS |
| CARD11 | RGPGPSVQHTTLNGDSLTSQLTLLGGNARGSFVHSVKPGSLAEKAGLREGHQLLL LEGCIRGERQSVPLDTCTKEEAHWTIQRCSGPVTLHYKVNHE |
| CARD14 | RRRPARRILSQVTMLAFQGDALLEQISVIGGNLTGIFIHRVTPGSAADQMALRPGT QIVMVDYEASEPLFKAVLEDTTLEEAVGLLRRVDGFCCLSVKVNTDGYKR |
| CASK | RVRLVQFQKNTDEPMGITLKMNELNHCIVARIMHGGMIHRQGTLHVGDEIREING ISVANQTVEQLQKMLREMRGSITFKIVPS |
| CNKSR1 | EQKAVLEQVQLDSPLGLEIHTTSNCQHFVSQVDTQVPTDSRLQIQPGDEVVQINEQ VVVREERDMVGWPRKNMVRELLREPAGLSLVLKK |
| CNKSR2 | SQSAHLEVIQLANIKPSEGLGMYIKSTYDGLHVITGTTENSPADRCKKIHAGDEVIQ VNHQTVVGWQLKNLVNALREDPSGVILTLKKRPQSMLTSAP |
| CNKSR3 | MSQCACLEEVHLPNIKPGEGLGMYIKSTYDGLHVITGTTENSPADRSQKIHAGDEV IQVNQQTVVGWQLKNLVKKLRENPTGVVLLLKKRPTGSFNFTPAP |
| DEPTOR (DEPDC6) | TPGAPYARKTFTIVGDAVGWGFVVRGSKPCHIQAVDPSGPAAAAGMKVCQFVVS VNGLNVLHVDYRTVSNLILTGPRTIVMEVMEELE |
| DFNB31_1 | GEVRLVSLRRAKAHEGLGFSIRGGSEHGVGIYVSLVEPGSLAEKEGLRVGDQILRV NDKSLARVTHAEAVKALKGSKKLVLSVYSAGRIPG |

| | |
|---|---|
| DFNB31_2 | GDRRSTLHLLQGGDEKKVNLVLGDGRSLGLTIRGGAEYGLGIYITGVDPGSEAEG SGLKVGDQILEVNGRSFLNILHDEAVRLLKSSRHLILTVKDVGRLPRARTTVDETK WIASSR |
| DFNB31_3 | TSTLVRVKKSAATLGIAIEGGANTRQPLPRIVTIQRGGSAHNCGQLKVGHVILEVN GLTLRGKEHREAARIIAEAFKTKDRDYIDFLVTEFN |
| DLG1_1 | EYEEITLERGNSGLGFSIAGGTDNPHIGDDSSIFITKIITGGAAAQDGRLRVNDCILR VNEVDVRDVTHSKAVEALKEAGSIVRLYVKRRKPVS |
| DLG1_2 | SEKIMEIKLIKGPKGLGFSIAGGVGNQHIPGDNSIYVTKIIEGGAAHKDGKLQIGDK LLAVNNVCLEEVTHEEAVTALKNTSDFVYLKVAKPTSMYMND |
| DLG1_3 | DDEITREPRKVVLHRGSTGLGFNIVGGEDGEGIFISFILAGGPADLSGELRKGDRIIS VNSVDLRAASHEQAAAALKNAGQAVTIVAQYRPEEYSRFEAKIHDLREQMMNSS ISS |
| DLG2_1 | EYEFEEITLERGNSGLGFSIAGGTDNPHIGDDPGIFITKIIPGGAAAEDGRLRVNDCI LRVNEVDVSEVSHSKAVEALKEAGSIVRLYVRRRRP |
| DLG2_2 | ETVVEIKLFKGPKGLGFSIAGGVGNQHIPGDNSIYVTKIIDGGAAQKDGRLQVGDR LLMVNNYSLEEVTHEEAVAILKNTSEVVYLKVGKPTTIY |
| DLG2_3 | EPRKVVLHKGSTGLGFNIVGGEDGEGIFVSFILAGGPADLSGELQRGDQILSVNGID LRGASHEQAAAALKGAGQTVTIIAQYQPEDYARFEAKIH |
| DLG3_1 | DGMFKYEEIVLERGNSGLGFSIAGGIDNPHVPDDPGIFITKIIPGGAAAMDGRLGVN DCVLRVNEVDVSEVVHSRAVEALKEAGPVVRLVVRRRQP |
| DLG3_2 | ETIMEVNLLKGPKGLGFSIAGGIGNQHIPGDNSIYITKIIEGGAAQKDGRLQIGDRLL AVNNTNLQDVRHEEAVASLKNTSDMVYLKVAKPGS |
| DLG3_3 | TREPRKIILHKGSTGLGFNIVGGEDGEGIFVSFILAGGPADLSGELRRGDRILSVNGV NLRNATHEQAAAALKRAGQSVTIVAQYRPEEYSRFESK |
| DLG4_1 | EYEEITLERGNSGLGFSIAGGTDNPHIGDDPSIFITKIIPGGAAAQDGRLRVNDSILFV NEVDVREVTHSAAVEALKEAGSIVRLYVMRRK |
| DLG4_2 | AEKVMEIKLIKGPKGLGFSIAGGVGNQHIPGDNSIYVTKIIEGGAAHKDGRLQIGD KILAVNSVGLEDVMHEDAVAALKNTYDVVYLKVAKPSNA |
| DLG4_3 | DIPREPRRIVIHRGSTGLGFNIVGGEDGEGIFISFILAGGPADLSGELRKGDQILSVNG VDLRNASHEQAAIALKNAGQTVTIIAQYKPEEYSRFEAK |
| DLG5_1 | ETEVVEFERETEDIDLKALGFDMAEGVNEPCFPGDCGIFVTKVDKGSIADGRLRVN DWLLRINDVDLINKDKKQAIKALLNGEGAINMVVRRRKSLG |
| DLG5_2 | GKVVTPLHINLSGQKDSGISLENGVYAAAVLPGSPAAKEGSLAVGDRIVAINGIAL DNKSLNECESLLRSCQDSLTLSLLKVFPQSS |
| DLG5_3 | GERRKDRPYVEEPRHVKVQKGSEPLGISIVSGEKGGIYVSKVTVGSIAHQAGLEYG DQLLEFNGINLRSATEQQARLIIGQQCDTITILAQYNPHVHQLSSHSRS |
| DLG5_4 | DANKKTLEPRVVFIKKSQLELGVHLCGGNLHGVFVAEVEDDSPAKGPDGLVPGD LILEYGSLDVRNKTVEEVYVEMLKPRDGVRLKVQYRPEEFTKAKGLPGDS |
| DVL1 | NIVTVTLNMERHHFLGISIVGQSNDRGDGGIYIGSIMKGGAVAADGRIEPGDMLLQ VNDVNFENMSNDDAVRVLREIVSQTGPISLTVAK |
| DVL2 | TMSLNIITVTLNMEKYNFLGISIVGQSNERGDGGIYIGSIMKGGAVAADGRIEPGD MLLQVNDMNFENMSNDDAVRVLRDIVHKPGPIVLTVAKCWDPSP |
| DVL3 | SLNIITVTLNMEKYNFLGISIVGQSNERGDGGIYIGSIMKGGAVAADGRIEPGDMLL QVNEINFENMSNDDAVRVLREIVHKPGPITLTVAKCWDPSPRG |

| | |
|---|---|
| FRMPD1 | PVRHTVKIDKDTLLQDYGFHISESLPLTVVAVTAGGSAHGKLFPGDQILQMNNEP AEDLSWERAVDILREAEDSLSITVVRCTSGVPKSS |
| FRMPD2_1 | GREIVRVTLKRDPHRGFGFVINEGEYSGQADPGIFISSIIPGGPAEKAKTIKPGGQIL ALNHISLEGFTFNMAVRMIQNSPDNIELIISQSK |
| FRMPD2_2 | SAGEIYFVELVKEDGTLGFSVTGGINTSVPYGGIYVKSIVPGGPAAKEGQILQGDRL LQVDGVILCGLTHKQAVQCLTGPGQVARLVLERRVPRS |
| FRMPD2_3 | TDGPKFEVKLKKNANGLGFSFVQMEKESCSHLKSDLVRIKRLFPGQPAEENGAIA AGDIILAVNGRSTEGLIFQEVLHLLRGAPQEVTLLLCRP |
| FRMPD3 | EQLPAEILRQVTVHRDPIYGFGFVAGSERPVVVRSVRPGGPSENKLLAGDQIVAIN EEDVSEAPRERLIELIRSAKEFIVLTVLHTHQS |
| FRMPD4 | ESCQIIPPAPRKVEMRRDPVLGFGFVAGSEKPVVVRSVTPGGPSEGKLIPGDQIVMI NDEPVSAAPRERVIDLVRSCKESILLTVIQPYPSPKS |
| GIPC1 | KGQRKEVEVFKSEDALGLTITDNGAGYAFIKRIKEGSVIDHIHLISVGDMIEAINGQ SLLGCRHYEVARLLKELPRGRTFTLKLTEPRKAFDMISQ |
| GIPC2 | KGIEKEVNVYKSEDSLGLTITDNGVGYAFIKRIKDGGVIDSVKTICVGDHIESINGE NIVGWRHYDVAKKLKELKKEELFTMKLIEPKKAFE |
| GIPC3 | RGETKEVEVTKTEDALGLTITDNGAGYAFIKRIKEGSIINRIEAVCVGDSIEAINDHS IVGCRHYEVAKMLRELPKSQPFTLRLVQPKRAFDMIGQ |
| GOPC | KKSQGVGPIRKVLLLKEDHEGLGISITGGKEHGVPILISEIHPGQPADRCGGLHVGD AILAVNGVNLRDTKHKEAVTILSQQRGEIEFEVVYVAPE |
| GORASP1 | MGLGVSAEQPAGGAEGFHLHGVQENSPAQQAGLEPYFDFIITIGHSRLNKENDTL KALLKANVEKPVKLEVFNMKTMRVREVEVVPSNMWGGQGLLGASVRFCSFRRA SEQVWHVLDVEPSSPAALAGLRPYTDYVVGSDQILQESEDFFTLIESHEGKPLKLM VYNSKSDSCREVTVTPNAAWGGEGSLGCGIGYGYLHRIPTQPPS |
| GORASP2 | GMGSSQSVEIPGGGTEGYHVLRVQENSPGHRAGLEPFFDFIVSINGSRLNKDNDTL KDLLKANVEKPVKMLIYSSKTLELRETSVTPSNLWGGQGLLGVSIRFCSFDGANE NVWHVLEVESNSPAALAGLRPHSDYIIGADTVMNESEDLFSLIETHEAKPLKLYVY NTDTDNCREVIITPNSAWGGEGSLGCGIGYGYLHRIPTRPFE |
| GRASP | EQQRKVLTLEKEDNQTFGFEIQTYGLHHREEQRVEMVTFVCRVHESSPAQLAGLT PGDTIASVNGLNVEGIRHREIVDIIKASGNVLRLETLYGTSIR |
| GRID2IP_1 | MATTATPATNQGWPEDFGFRLGGSGPCFVLEVAKGSSAHAGGLRPGDQILEVEGL AVGGLSRERLVRLARRCPRVPPSLGVLPAPDG |
| GRID2IP_2 | GPGGARRTVRVYKGNKSFGFTLRGHGPVWIESVLPGSPADNAALKSGDRILFLNG LDMRNCSHDKVVSMLQGSGAMPTLVVEEGLVPFASDSDSLDSPN |
| GRIP1_1 | EFKGSTVVELMKKEGTTLGLTVSGGIDKDGKPRVSNLRQGGIAARSDQLDVGDYI KAVNGINLAKFRHDEIISLLKNVGERVVLEVEYELP |
| GRIP1_2 | SVIFRTVEVTLHKEGNTFGFVIRGGAHDDRNKSRPVVITCVRPGGPADREGTIKPG DRLLSVDGIRLLGTTHAEAMSILKQCGQEAALLIEYDVS |
| GRIP1_3 | DSVATASGPLLVEVAKTPGASLGVALTTSMCCNKQVIVIDKIKSASIADRCGALHV GDHILSIDGTSMEYCTLAEATQFLANTTDQVKLEILPHHQTRLALKG |
| GRIP1_4 | QVVHTETTEVVLTADPVTGFGIQLQGSVFATETLSSPPLISYIEADSPAERCGVLQI GDRVMAINGIPTEDSTFEEASQLLRDSSITSKVTLEIEFDVAESVIP |
| GRIP1_5 | ESVIPSSGTFHVKLPKKHNVELGITISSPSSRKPGDPLVISDIKKGSVAHRTGTLELG DKLLAIDNIRLDNCSMEDAVQILQQCEDLVKLKIRKDED |

| | |
|---|---|
| GRIP1_6 | SSGAIIYTVELKRYGGPLGITISGTEEPFDPIIISSLTKGGLAERTGAIHIGDRILAINSS SLKGKPLSEAIHLLQMAGETVTLKIKKQTDAQSASS |
| GRIP1_7 | SPTPVELHKVTLYKDSDMEDFGFSVADGLLEKGVYVKNIRPAGPGDLGGLKPYD RLLQVNHVRTRDFDCCLVVPLIAESGNKLDLVISRNP |
| GRIP2_1 | EEFRGITVVELIKKEGSTLGLTISGGTDKDGKPRVSNLRPGGLAARSDLLNIGDYIR SVNGIHLTRLRHDEIITLLKNVGERVVLEVEYELPP |
| GRIP2_2 | ENNPRIISKTVDVSLYKEGNSFGFVLRGGAHEDGHKSRPLVLTYVRPGGPADREGS LKVGDRLLSVDGIPLHGASHATALATLRQCSHEALFQVEYDVATPDTVANASG |
| GRIP2_3 | DTVANASGPLMVEIVKTPGSALGISLTTTSLRNKSVITIDRIKPASVVDRSGALHPG DHILSIDGTSMEHCSLLEATKLLASISEKVRLEILPVPQSQRPLR |
| GRIP2_4 | GGQIVHTETTEVVLCGDPLSGFGLQLQGGIFATETLSSPPLVCFIEPDSPAERCGLL QVGDRVLSINGIATEDGTMEEANQLLRDAALAHKVVLEVEFDVAES |
| GRIP2_5 | DVAESVIPSSGTFHVKLPKKRSVELGITISSASRKRGEPLIISDIKKGSVAHRTGTLEP GDKLLAIDNIRLDNCPMEDAVQILRQCEDLVKLKIRKDED |
| GRIP2_6 | TTGAVSYTVELKRYGGPLGITISGTEEPFDPIVISGLTKRGLAERTGAIHVGDRILAI NNVSLKGRPLSEAIHLLQVAGETVTLKIKKQLDR |
| GRIP2_7 | PTPLEMHKVTLHKDPMRHDFGFSVSDGLLEKGVYVHTVRPDGPAHRGGLQPFDR VLQVNHVRTRDFDCCLAVPLLAEAGDVLELIISRKP |
| HTRA1 | TESHDRQAKGKAITKKKYIGIRMMSLTSSKAKELKDRHRDFPDVISGAYIIEVIPDT PAEAGGLKENDVIISINGQSVVSANDVSDVIKRESTLNMVVRRGNEDIMITVIPEEI DP |
| HTRA2 | SGSQRRYIGVMMLTLSPSILAELQLREPSFPDVQHGVLIHKVILGSPAHRAGLRPGD VILAIGEQMVQNAEDVYEAVRTQSQLAVQIRRGRETLTLYVTPEVTE |
| HTRA3 | KKRFIGIRMRTITPSLVDELKASNPDFPEVSSGIYVQEVAPNSPSQRGGIQDGDIIVK VNGRPLVDSSELQEAVLTESPLLLEVRRGNDDLLFSIAPEVVM |
| HTRA4 | HQMKGKAFSNKKYLGLQMLSLTVPLSEELKMHYPDFPDVSSGVYVCKVVEGTA AQSSGLRDHDVIVNINGKPITTTTDVVKALDSDSLSMAVLRGKDNLLLTVIPETIN |
| IL16_1 | QASVISNIVLMKGQAKGLGFSIVGGKDSIYGPIGIYVKTIFAGGAAAADGRLQEGD EILELNGESMAGLTHQDALQKFKQAKKGLLTLTVRTRLTAP |
| IL16_2 | STAKPNYRIMVEVSLQKEAGVGLGIGLCSVPYFQCISGIFVHTLSPGSVAHLDGRL RCGDEIVEISDSPVHCLTLNEVYTILSHCDPGPVPIIVSRHPDPQVSEQQLKE |
| IL16_3 | KQLDGIHVTILHKEEGAGLGFSLAGGADLENKVITVHRVFPNGLASQEGTIQKGNE VLSINGKSLKGTTHHDALAILRQAREPRQAVIVTRKLTPE |
| IL16_4 | MPDLNSSTDSAASASAASDVSVESTAEATVCTVTLEKMSAGLGFSLEGGKGSLHG DKPLTINRIFKGAASEQSETVQPGDEILQLGGTAMQGLTRFEAWNIIKALPDGPVTI VIRRKSLQSKETTAAGDS |
| InaDl_1 | KLGNEDFNSVIQQMAQGRQIEYIDIERPSTGGLGFSVVALRSQNLGKVDIFVKDVQ PGSVADRDQRLKENDQILAINHTPLDQNISHQQAIALLQQTTGSLRLIVAREPVHT KSSTS |
| InaDl_10 | EPRTVEINRELSDALGISIAGGRGSPLGDIPVFIAMIQASGVAARTQKLKVGDRIVSI NGQPLDGLSHADVVNLLKNAYGRIILQVVADTN |
| InaDl_2 | PETVCWGHVEEVELINDGSGLGFGIVGGKTSGVVVRTIVPGGLADRDGRLQTGDH ILKIGGTNVQGMTSEQVAQVLRNCGNSVRMLVARDPAGDISVT |

| | |
|---|---|
| InaDl_3 | SLFETYNVELVRKDGQSLGIRIVGYVGTSHTGEASGIYVKSIIPGSAAYHNGHIQVN DKIVAVDGVNIQGFANHDVVEVLRNAGQVVHLTLVRRKTSSSTSPLEPPSDRGT |
| InaDl_4 | DTQIADDAELQKYSKLLPIHTLRLGVEVDSFDGHHYISSIVSGGPVDTLGLLQPEDE LLEVNGMQLYGKSRREAVSFLKEVPPPFTLVCCRRLFDDEASVDEPRR |
| InaDl_5 | DDGELALWSPEVKIVELVKDCKGLGFSILDYQDPLDPTRSVIVIRSLVADGVAERS GGLLPGDRLVSVNEYCLDNTSLAEAVEILKAVPPGLVHLGICKPLVEDNEEE |
| InaDl_6 | PNFSHWGPPRIVEIFREPNVSLGISIVGGQTVIKRLKNGEELKGIFIKQVLEDSPAGK TNALKTGDKILEVSGVDLQNASHSEAVEAIKNAGNPVVFIVQSLSSTPRVIP |
| InaDl_7 | DAFTDQKIRQRYADLPGELHIIELEKDKNGLGLSLAGNKDRSRMSIFVVGINPEGP AAADGRMRIGDELLEINNQILYGRSHQNASAIIKTAPSKVKLVFIRNEDAVNQMA VTP |
| InaDl_8 | PATCPIVPGQEMIIEISKGRSGLGLSIVGGKDTPLNAIVIHEVYEEGAAARDGRLWA GDQILEVNGVDLRNSSHEEAITALRQTPQKVRLVVYRDEAHYRDEENLE |
| InaDl_9 | EIFPVDLQKKAGRGLGLSIVGKRNGSGVFISDIVKGGAADLDGRLIQGDQILSVNG EDMRNASQETVATILKCAQGLVQLEIGRLR |
| INTU | KEQLKLLEVLVGIIHQTKWSWRRTGKQGDGERLVVHGLLPGGSAMKSGQVLIGD VLVAVNDVDVTTENIERVLSCIPGPMQVKLTFENAYDVKRETSHPRQK |
| LAP2 (Erbin) | GHELAKQEIRVRVEKDPELGFSISGGVGGRGNPFRPDDDGIFVTRVQPEGPASKLL QPGDKIIQANGYSFINIEHGQAVSLLKTFQNTVELIIVREVSS |
| LDB3 | MSYSVTLTGPGPWGFRLQGGKDFNMPLTISRITPGSKAAQSQLSQGDLVVAIDGV NTDTMTHLEAQNKIKSASYNLSLTLQKSKR |
| LIMK1 | PGSHLPHTVTLVSIPASSHGKRGLSVSIDPPHGPPGCGTEHSHTVRVQGVDPGCMS PDVKNSIHVGDRILEINGTPIRNVPLDEIDLLIQETSRLLQLTLEHDPHDTLGHGLGP |
| LIMK2 | QEQLPYSVTLISMPATTEGRRGFSVSVESACSNYATTVQVKEVNRMHISPNNRNAI HPGDRILEINGTPVRTLRVEEVEDAISQTSQTLQLLIEHDPVSQRLDQLR |
| LIN7A | SEGHSHPRVVELPKTDEGLGFNVMGGKEQNSPIYISRIIPGGVAERHGGLKRGDQL LSVNGVSVEGEHHEKAVELLKAAKDSVKLVVRYTPK |
| LIN7B | SEGHAHPRVVELPKTDEGLGFNIMGGKEQNSPIYISRVIPGGVADRHGGLKRGDQ LLSVNGVSVEGEQHEKAVELLKAAQGSVKLVVRYTPR |
| LIN7C | SEGHSHPRVVELPKTEEGLGFNIMGGKEQNSPIYISRIIPGGIADRHGGLKRGDQLL SVNGVSVEGEHHEKAVELLKAAQGKVKLVVRYTPK |
| LMO7 | QFSDMRISINQTPGKSLDFGFTIKWDIPGIFVASVEAGSPAEFSQLQVDDEIIAINNT KFSYNDSKEWEEAMAKAQETGHLVMDVRRYGK |
| LNX1_1 | PRLYHLIPDGEITSIKINRVDPSESLSIRLVGGSETPLVHIIIQHIYRDGVIARDGRLLP GDIILKVNGMDISNVPHNYAVRLLRQPCQVLWLTVMREQKFRSRNNGQAPD |
| LNX1_2 | DAYRPRDDSFHVILNKSSPEEQLGIKLVRKVDEPGVFIFNVLDGGVAYRHGQLEEN DRVLAINGHDLRYGSPESAAHLIQASERRVHLVVSRQVRQRSPD |
| LNX1_3 | TITCHEKVVNIQKDPGESLGMTVAGGASHREWDLPIYVISVEPGGVISRDGRIKTG DILLNVDGVELTEVSRSEAVALLKRTSSSIVLKALEVKEYE |
| LNX1_4 | RCLYNCKDIVLRRNTAGSLGFCIVGGYEEYNGNKPFFIKSIVEGTPAYNDGRIRCG DILLAVNGRSTSGMIHACLARLLKELKGRITLTIVSWPGT |

| | |
|---|---|
| LNX2_1 | PLSLPEGEITTIEIHRSNPYIQLGISIVGGNETPLINIVIQEVYRDGVIARDGRLLAGD<br>QILQVNNYNISNVSHNYARAVLSQPCNTLHLTVLRERRFGNRAHN |
| LNX2_2 | REEIFQVALHKRDSGEQLGIKLVRRTDEPGVFILDLLEGGLAAQDGRLSSNDRVLA<br>INGHDLKYGTPELAAQIIQASGERVNLTIARPGKPQP |
| LNX2_3 | TQCVTCQEKHITVKKEPHESLGMTVAGGRGSKSGELPIFVTSVPPHGCLARDGRIK<br>RGDVLLNINGIDLTNLSHSEAVAMLKASAASPAVALKALEVQIVEE |
| LNX2_4 | PSTLHSCHDIVLRRSYLGSWGFSIVGGYEENHTNQPFFIKTIVLGTPAYYDGRLKC<br>GDMIVAVNGLSTVGMSHSALVPMLKEQRNKVTLTVICWPGS |
| LRRC7 | EQFCVRIEKNPGLGFSISGGISGQGNPFKPSDKGIFVTRVQPDGPASNLLQPGDKIL<br>QANGHSFVHMEHEKAVLLLKSFQNTVDLVIQRELT |
| MAGI1_1 | MSKVIQKKNHWTSRVHECTVKRGPQGELGVTVLGGAEHGEFPYVGAVAAVEAA<br>GLPGGGEGPRLGEGELLLEVQGVRVSGLPRYDVLGVIDSCKEAVTFKAVRQGG |
| MAGI1_2 | KPFFTRNPSELKGKFIHTKLRKSSRGFGFTVVGGDEPDEFLQIKSLVLDGPAALDG<br>KMETGDVIVSVNDTCVLGHTHAQVVKIFQSIPIGASVDLELCRGYPLPFDPDDPNT<br>SLVTSVAILDKEP |
| MAGI1_3 | QPELITVHIVKGPMGFGFTIADSPGGGGQRVKQIVDSPRCRGLKEGDLIVEVNKKN<br>VQALTHNQVVDMLVECPKGSEVTLLVQRGGLP |
| MAGI1_4 | DYQEQDIFLWRKETGFGFRILGGNEPGEPIYIGHIVPLGAADTDGRLRSGDELICVD<br>GTPVIGKSHQLVVQLMQQAAKQGHVNLTVRRKVVSGP |
| MAGI1_5 | GSGVVSTVVQPYDVEIRRGENEGFGFVIVSSVSRPEAGTTFGNACVAMPHKIGRIIE<br>GSPADRCGKLKVGDRILAVNGCSITNKSHSDIVNLIKEAGNTVTLRIIPGDESSN |
| MAGI1_6 | QEQDFYTVELERGAKGFGFSLRGGREYNMDLYVLRLAEDGPAERCGKMRIGDEI<br>LEINGETTKNMKHSRAIELIKNGGRRVRLFLKRGDGSVP |
| MAGI2_1 | MSKSLKKKSHWTSKVHESVIGRNPEGQLGFELKGGAENGQFPYLGEVKPGKVAY<br>ESGSKLVSEELLLEVNETPVAGLTIRDVLAVIKHCKDPLRLKCVKQGG |
| MAGI2_2 | KPLFTRDASQLKGTFLSTTLKKSNMGFGFTIIGGDEPDEFLQVKSVIPDGPAAQDG<br>KMETGDVIVYINEVCVLGHTHADVVKLFQSVPIGQSVNLVLCRGYPLPFDPEDPA<br>NS |
| MAGI2_3 | QAELMTLTIVKGAQGFGFTIADSPTGQRVKQILDIQGCPGLCEGDLIVEINQQNVQ<br>NLSHTEVVDILKDCPIGSETSLIIHRG |
| MAGI2_4 | PDYKELDVHLRRMESGFGFRILGGDEPGQPILIGAVIAMGSADRDGRLHPGDELV<br>YVDGIPVAGKTHRYVIDLMHHAARNGQVNLTVRRKVLCG |
| MAGI2_5 | SLQTSDVVIHRKENEGFGFVIISSLNRPESGSTITVPHKIGRIIDGSPADRCAKLKVG<br>DRILAVNGQSIINMPHADIVKLIKDAGLSVTLRIIPQEELNSPTS |
| MAGI2_6 | QDFDYFTVDMEKGAKGFGFSIRGGREYKMDLYVLRLAEDGPAIRNGRMRVGDQI<br>IEINGESTRDMTHARAIELIKSGGRRVRLLLKRGT |
| MAGI3_1 | MSKTLKKKKHWLSKVQECAVSWAGPPGDFGAEIRGGAERGEFPYLGRLREEPGG<br>GTCCVVSGKAPSPGDVLLEVNGTPVSGLTNRDTLAVIRHFREPIRLKTVKPGKVIN<br>KDLR |
| MAGI3_2 | TRDPSQLKGVLVRASLKKSTMGFGFTIIGGDRPDEFLQVKNVLKDGPAAQDGKIA<br>PGDVIVDINGNCVLGHTHADVVQMFQLVPVNQYVNLTLCRGYPLPDDSEDP |
| MAGI3_3 | SQPELVTIPLIKGPKGFGFAIADSPTGQKVKMILDSQWCQGLQKGDIIKEIYHQNVQ<br>NLTHLQVVEVLKQFPVGADVPLLILRG |
| MAGI3_4 | EDKPPNTKDLDVFLRKQESGFGFRVLGGDGPDQSIYIGAIIPLGAAEKDGRLRAAD<br>ELMCIDGIPVKGKSHKQVLDLMTTAARNGHVLLTVRRKIFYGEK |

| | |
|---|---|
| MAGI3_5 | QEPYDVVLQRKENEGFGFVILTSKNKPPPGVIPHKIGRVIEGSPADRCGKLKVGDHISAVNGQSIVELSHDNIVQLIKDAGVTVTLTVIAEEEHH |
| MAGI3_6 | NQNLGCYPVELERGPRGFGFSLRGGKEYNMGLFILRLAEDGPAIKDGRIHVGDQIVEINGEPTQGITHTRAIELIQAGGNKVLLLLRPGT |
| MAGIX | SQASGHFSVELVRGYAGFGLTLGGGRDVAGDTPLAVRGLLKDGPAQRCGRLEVGDVVLHINGESTQGLTHAQAVERIRAGGPQLHLVIRRPLET |
| MAST1 | RSPITIQRSGKKYGFTLRAIRVYMGDTDVYSVHHIVWHVEEGGPAQEAGLCAGDLITHVNGEPVHGMVHPEVVELILKSGNKVAVTTTPFEN |
| MAST2 | RPPIIIHRAGKKYGFTLRAIRVYMGDSDVYTVHHMVWHVEDGGPASEAGLRQGDLITHVNGEPVHGLVHTEVVELILKSGNKVAISTTPLEN |
| MAST3 | RPPIVIHSSGKKYGFSLRAIRVYMGDSDVYTVHHVVWSVEDGSPAQEAGLRAGDLITHINGESVLGLVHMDVVELLLKSGNKISLRTTALEN |
| MAST4 | QPIVIHSSGKNYGFTIRAIRVYVGDSDIYTVHHIVWNVEEGSPACQAGLKAGDLITHINGEPVHGLVHTEVIELLLKSGNKVSITTTPFENTS |
| MLLT4 | RKEPEIITVTLKKQNGMGLSIVAAKGAGQDKLGIYVKSVVKGGAADVDGRLAAGDQLLSVDGRSLVGLSQERAAELMTRTSSVVTLEVAKQGAIYH |
| MPDZ_1 | DEFDQLIKNMAQGRHVEVFELLKPPSGGLGFSVVGLRSENRGELGIFVQEIQEGSVAHRDGRLKETDQILAINGQALDQTITHQQAISILQKAKDTVQLVIARGSLPQLVS |
| MPDZ_10 | PGCETTIEISKGRTGLGLSIVGGSDTLLGAIIIHEVYEEGAACKDGRLWAGDQILEVNGIDLRKATHDEAINVLRQTPQRVRLTLYRDEAPYK |
| MPDZ_11 | DTLTIELQKKPGKGLGLSIVGKRNDTGVFVSDIVKGGIADADGRLMQGDQILMVNGEDVRNATQEAVAALLKCSLGTVTLEVGRIKAGP |
| MPDZ_12 | QGLRTVEMKKGPTDSLGISIAGGVGSPLGDVPIFIAMMHPTGVAAQTQKLRVGDRIVTICGTSTEGMTHTQAVNLLKNASGSIEMQVVAGGD |
| MPDZ_13 | PPQCKSITLERGPDGLGFSIVGGYGSPHGDLPIYVKTVFAKGAASEDGRLKRGDQIIAVNGQSLEGVTHEEAVAILKRTKGTVTLMVLS |
| MPDZ_2 | HSNPVHWQHMETIELVNDGSGLGFGIIGGKATGVIVKTILPGGVADQHGRLCSGDHILKIGDTDLAGMSSEQVAQVLRQCGNRVKLMIARGAIEERT |
| MPDZ_3 | EESETFDVELTKNVQGLGITIAGYIGDKKLEPSGIFVKSITKSSAVEHDGRIQIGDQIIAVDGTNLQGFTNQQAVEVLRHTGQTVLLTLMRRGMKQE |
| MPDZ_4 | NYEIVVAHVSKFSENSGLGISLEATVGHHFIRSVLPEGPVGHSGKLFSGDELLEVNGITLLGENHQDVVNILKELPIEVTMVCCRRT |
| MPDZ_5 | QAPLAMWEAGIQHIELEKGSKGLGFSILDYQDPIDPASTVIIIRSLVPGGIAEKDGRLLPGDRLMFVNDVNLENSSLEEAVEALKGAPSGTVRIGVAKPLPLSPEE |
| MPDZ_6 | QNVSKESFERTINIAKGNSSLGMTVSANKDGLGMIVRSIIHGGAISRDGRIAIGDCILSINEESTISVTNAQARAMLRRHSLIGPDIKITYVPAEHLEEFKISLGQQS |
| MPDZ_7 | TAYSNWNQPRRVELWREPSKSLGISIVGGRGMGSRLSNGEVMRGIFIKHVLEDSPAGKNGTLKPGDRIVEVDGMDLRDASHEQAVEAIRKAGNPVVFMVQSIINRP |
| MPDZ_8 | DKEDEFGYSWKNIRERYGTLTGELHMIELEKGHSGLGLSLAGNKDRSRMSVFIVGIDPNGAAGKDGRLQIADELLEINGQILYGRSHQNASSIIKCAPSKVKIIFIRNKDAVNQMAV |
| MPDZ_9 | PTVTTSDAAVDLSSFKNVQHLELPKDQGGLGIAISEEDTLSGVIIKSLTEHGVAATDGRLKVGDQILAVDDEIVVGYPIEKFISLLKTAKMTVKLTIHAENPDSQAVPS |

| | |
|---|---|
| MPP1 | VRLIQFEKVTEEPMGITLKLNEKQSCTVARILHGGMIHRQGSLHVGDEILEINGTN VTNHSVDQLQKAMKETKGMISLKVIPNQQ |
| MPP2 | DAVRMVGIRKTAGEHLGVTFRVEGGELVIARILHGGMVAQQGLLHVGDIIKEVN GQPVGSDPRALQELLRNASGSVILKILPSYQE |
| MPP3 | DNIDEDFDEESVKIVRLVKNKEPLGATIRRDEHSGAVVVARIMRGGAADRSGLVH VGDELREVNGIAVLHKRPDEISQILAQSQGSITLKIIPATQEED |
| MPP4 | PDNIPESEEAMRIVCLVKNQQPLGATIKRHEMTGDILVARIIHGGLAERSGLLYAG DKLVEVNGVSVEGLDPEQVIHILAMSRGTIMFKVVPVSDPPVNS |
| MPP5 | TDERVYESIGQYGGETVKIVRIEKARDIPLGATVRNEMDSVIISRIVKGGAAEKSGL LHEGDEVLEINGIEIRGKDVNEVFDLLSDMHGTLTFVLIPSQ |
| MPP6 | DAIRILGIHKRAGEPLGVTFRVENNDLVIARILHGGMIDRQGLLHVGDIIKEVNGHE VGNNPKELQELLKNISGSVTLKILPS |
| MPP7 | DPVLPPMPEDIDDEEDSVKIIRLVKNREPLGATIKKDEQTGAIIVARIMRGGAADRS GLIHVGDELREVNGIPVEDKRPEEIIQILAQSQGAITFKIIPGSKEET |
| MYO18A | TLRELELQRRPTGDFGFSLRRTTMLDRGPEGQACRRVVHFAEPGAGTKDLALGLV PGDRLVEINGHNVESKSRDEIVEMIRQSGDSVRLKVQP |
| NHERF_1 (SLC9A3R1) | PLPRLCCLEKGPNGYGFHLHGEKGKLGQYIRLVEPGSPAEKAGLLAGDRLVEVNG ENVEKETHQQVVSRIRAALNAVRLLVVDPETDEQ |
| NHERF_2 | EQRELRPRLCTMKKGPSGYGFNLHSDKSKPGQFIRSVDPDSPAEASGLRAQDRIVE VNGVCMEGKQHGDVVSAIRAGGDETKLLVVDRETDE |
| NHERF2_1 (SLC9A3R2) | PRLCRLVRGEQGYGFHLHGEKGRRGQFIRRVEPGSPAEAAALRAGDRLVEVNGV NVEGETHHQVVQRIKAVEGQTRLLVVDQE |
| NHERF2_2 | GPLRELRPRLCHLRKGPQGYGFNLHSDKSRPGQYIRSVDPGSPAARSGLRAQDRLI EVNGQNVEGLRHAEVVASIKAREDEARLLVVDPETDEHFKR |
| NHERF3_1 (PDZK1) | TSTFNPRECKLSKQEGQNYGFFLRIEKDTEGHLVRVVEKCSPAEKAGLQDGDRVL RINGVFVDKEEHMQVVDLVRKSGNSVTLLVLDGDSYEKAVKTRVDLKELGQ |
| NHERF3_2 | QPRLCYLVKEGGSYGFSLKTVQGKKGVYMTDITPQGVAMRAGVLADDHLIEVNG ENVEDASHEEVVEKVKKSGSRVMFLLVDKETDKRHVEQK |
| NHERF3_3 | PHQPRIVEMKKGSNGYGFYLRAGSEQKGQIIKDIDSGSPAEEAGLKNNDLVVAVN GESVETLDHDSVVEMIRKGGDQTSLLVVDKETDNMYR |
| NHERF3_4 | KPKLCRLAKGENGYGFHLNAIRGLPGSFIKEVQKGGPADLAGLEDEDVIIEVNGV NVLDEPYEKVVDRIQSSGKNVTLLVCGKKA |
| NHERF4_1 (PDZD3) | DPYDPWSLERPRFCLLSKEEGKSFGFHLQQELGRAGHVVCRVDPGTSAQRQGLQE GDRILAVNNDVVEHEDYAVVVRRIRASSPRVLLTVLARHAHDVARAQLGED |
| NHERF4_2 | RPRLCHIVKDEGGFGFSVTHGNQGPFWLVLSTGGAAERAGVPPGARLLEVNGVS VEKFTHNQLTRKLWQSGQQVTLLVAGPEVEEQCR |
| NHERF4_3 | TKPRCLHLEKGPQGFGFLLREEKGLDGRPGQFLWEVDPGLPAKKAGMQAGDRLV AVAGESVEGLGHEETVSRIQGQGSCVSLTVVDPE |
| NHERF4_4 | GSRQCFLYPGPGGSYGFRLSCVASGPRLFISQVTPGGSAARAGLQVGDVILEVNGY PVGGQNDLERLQQLPEAEPPLCLKLAARSLR |
| NOS1 | QQIQPNVISVRLFKRKVGGLGFLVKERVSKPPVIISDLIRGGAAEQSGLIQAGDIILA VNGRPLVDLSYDSALEVLRGIASETHVVLILRGPE |

| | |
|---|---|
| PARD3_1 | PNFSLDDMVKLVEVPNDGGPLGIHVVPFSARGGRTLGLLVKRLEKGGKAEHENLFRENDCIVRINDGDLRNRRFEQAQHMFRQAMRTPIIWFHVVPAANKEQYEQLSQS |
| PARD3_2 | KKIGKRLNIQLKKGTEGLGFSITSRDVTIGGSAPIYVKNILPRGAAIQDGRLKAGDRLIEVNGVDLVGKSQEEVVSLLRSTKMEGTVSLLVFRQEDA |
| PARD3_3 | GTREFLTFEVPLNDSGSAGLGVSVKGNRSKENHADLGIFVKSIINGGAASKDGRLRVNDQLIAVNGESLLGKTNQDAMETLRRSMSTEGNKRGMIQLIVARRIS |
| PARD3B_1 | QTELLTSPRTKDTLSDMTRTVEISGEGGPLGIHVVPFFSSLSGRILGLFIRGIEDNSRSKREGLFHENECIVKINNVDLVDKTFAQAQDVFRQAMKSPSVLLHVLPPQNREQYEKS |
| PARD3B_2 | NKNAKKIKIDLKKGPEGLGFTVVTRDSSIHGPGPIFVKNILPKGAAIKDGRLQSGDRILEVNGRDVTGRTQEELVAMLRSTKQGETASLVIARQEG |
| PARD3B_3 | ETSEQLTFEIPLNDSGSAGLGVSLKGNKSRETGTDLGIFIKSIIHGGAAFKDGRLRMNDQLIAVNGESLLGKSNHEAMETLRRSMSMEGNIRGMIQLVILRRPER |
| PARD6A | PETHRRVRLHKHGSDRPLGFYIRDGMSVRVAPQGLERVPGIFISRLVRGGLAESTGLLAVSDEILEVNGIEVAGKTLDQVTDMMVANSHNLIVTVKPANQR |
| PARD6B | DFRPVSSIIDVDILPETHRRVRLYKYGTEKPLGFYIRDGSSVRVTPHGLEKVPGIFISRLVPGGLAQSTGLLAVNDEVLEVNGIEVSGKSLDQVTDMMIANSRNLIITVRPANQR |
| PARD6G | RPVSSIIDVDLVPETHRRVRLHRHGCEKPLGFYIRDGASVRVTPHGLEKVPGIFISRMVPGGLAESTGLLAVNDEVLEVNGIEVAGKTLDQVTDMMIANSHNLIVTVKPANQRN |
| PCLO | NGKTMHYIFPHARIKITRDSKDHTVSGNGLGIRIVGGKEIPGHSGEIGAYIAKILPGGSAEQTGKLMEGMQVLEWNGIPLTSKTYEEVQSIISQQSGEAEICVRLDLNMLSDSEN |
| PDLIM1 | MTTQQIDLQGPGPWGFRLVGGKDFEQPLAISRVTPGSKAALANLCIGDVITAIDGENTSNMTHLEAQNRIKGCTDNLTLTVARSEHK |
| PDLIM2 | MALTVDVAGPAPWGFRITGGRDFHTPIMVTKVAERGKAKDADLRPGDIIVAINGESAEGMLHAEAQSKIRQSPSPLRLQLDRS |
| PDLIM3 | MPQTVILPGPAPWGFRLSGGIDFNQPLVITRITPGSKAAAANLCPGDVILAIDGFGTESMTHADAQDRIKAAAHQLCLKIDRGETHLWSPQVSE |
| PDLIM4 | MPHSVTLRGPSPWGFRLVGGRDFSAPLTISRVHAGSKAALAALCPGDLIQAINGESTELMTHLEAQNRIKGCHDHLTLSVSRP |
| PDLIM5 | MSNYSVSLVGPAPWGFRLQGGKDFNMPLTISSLKDGGKAAQANVRIGDVVLSIDGINAQGMTHLEAQNKIKGCTGSLNMTLQRASAAPKPEP |
| PDLIM7 | MDSFKVVLEGPAPWGFRLQGGKDFNVPLSISRLTPGGKAAQAGVAVGDWVLSIDGENAGSLTHIEAQNKIRACGERLSLGLSRAQ |
| PDZD11 | NNELTQFLPRTITLKKPPGAQLGFNIRGGKASQLGIFISKVIPDSDAHRAGLQEGDQVLAVNDVDFQDIEHSKAVEILKTAREISMRVRFFP |
| PDZD2_1 | PEMEICTVYLTKELGDTETVGLSFGNIPVFGDYGEKRRGGKKRKTHQGPVLDVGCIWVTELRKNSPAGKSGKVRLRDEILSLNGQLMVGVDVSGASYLAEQCWNGGFIYLIMLRRFKH |
| PDZD2_2 | REEVGRIWKMELLKESDGLGIQVSGGRGSKRSPHAIVVTQVKEGGAAHRDGRLSLGDELLVINGHLLVGLSHEEAVAILRSATGMVQLVVASKENSAED |

| | |
|---|---|
| PDZD2_3 | PWRLIRPSVISIIGLYKEKGKGLGFSIAGGRDCIRGQMGIFVKTIFPNGSAAEDGRLK EGDEILDVNGIPIKGLTFQEAIHTFKQIRSGLFVLTVRTKLVSPSLTPCSTP |
| PDZD2_4 | KDRIVMEVTLNKEPRVGLGIGACCLALENSPPGIYIHSLAPGSVAKMESNLSRGDQ ILEVNSVNVRHAALSKVHAILSKCPPGPVRLVIGRHPN |
| PDZD2_5 | KAQSENEEDVCFIVLNRKEGSGLGFSVAGGTDVEPKSITVHRVFSQGAASQEGTM NRGDFLLSVNGASLAGLAHGNVLKVLHQAQLHKDALVVIKKGMDQPRPSARQE |
| PDZD2_6 | RSVAVHDALCVEVLKTSAGLGLSLDGGKSSVTGDGPLVIKRVYKGGAAEQAGIIE AGDEILAINGKPLVGLMHFDAWNIMKSVPEGPVQLLIRKHRNSS |
| PDZD4 | PQEADRLDELEYEEVELYKSSHRDKLGLMVCYRTDDEEDLGIYVGEVNPNSIAAK DGRIREGDRIIQINGVDVQNREEAVAILSQEENTNISLLVARPESQLAKRWKDSD |
| PDZD7_1 | DIIHSVRVEKSPAGRLGFSVRGGSEHGLGIFVSKVEEGSSAERAGLCVGDKITEVN GLSLESTTMGSAVKVLTSSSRLHMMVRRMGRVP |
| PDZD7_2 | SDTSSEDGVRRIVHLYTTSDDFCLGFNIRGGKEFGLGIYVSKVDHGGLAEENGIKV GDQVLAANGVRFDDISHSQAVEVLKGQTHIMLTIKETGRYPAYKEMVSEYCWLD RLSNG |
| PDZD7_3 | GELKTVTLSKMKQSLGISISGGIESKVQPMVKIEKIFPGGAAFLSGALQAGFELVAV DGENLEQVTHQRAVDTIRRAYRNKAREPMELVVRVPGPS |
| PDZD8 | KQRSSIKTVELIKGNLQSVGLTLRLVQSTDGYAGHVIIETVAPNSPAAIADLQRGD RLIAIGGVKITSTLQVLKLIKQAGDRVLVYYERPVGQSNQGAVLQDN |
| PDZD9 | HNLSKTQQTKLTVGSLGLGLIIIQHGPYLQITHLIRKGAAANDGKLQPGDVLISVG HANVLGYTLREFLQLLQHITIGTVLQIKVYRDFINIPEEWQE |
| PDZRN3_1 | TKSLTLVLHRDSGSLGFNIIGGRPSVDNHDGSSSEGIFVSKIVDSGPAAKEGGLQIH DRIIEVNGRDLSRATHDQAVEAFKTAKEPIVVQVLRRT |
| PDZRN3_2 | DIHQEMDREELELEEVDLYRMNSQDKLGLTVCYRTDDEDDIGIYISEIDPNSIAAK DGRIREGDRIIQINGIEVQNREEAVALLTSEENKNFSLLIARPELQLDEGWMDDD |
| PDZRN4_1 | HRRDGEHKPFTIVLERENDTLGFNIIGGRPNQNNQEGTSTEGIYVSKILENGPADRA DGLEIHDKIMEVNGKDLSKATHEEAVEAFRNAKEPIVVQVLRRTPLSR |
| PDZRN4_2 | PADADRTEDFEYEEVELCRVSSQEKLGLTVCYRTDDEEDTGIYVSEVDPNSIAAKD GRIREGDRILQINGEDVQNREEAVALLSNDECKRIVLLVARPEIQLDEGWLEDE |
| PICK1 | TVPGKVTLQKDAQNLIGISIGGGAQYCPCLYIVQVFDNTPAALDGTVAAGDEITGV NGRSIKGKTKVEVAKMIQEVKGEVTIHYNKLQADPKQ |
| PPP1R9A | KLELFPVELEKDEDGLGISIIGMGVGADAGLEKLGIFVKTVTEGGAAQRDGRIQVN DQIVEVDGISLVGVTQNFAATVLRNTKGNVRFVIGREKP |
| PPP1R9B | RLELFPVELEKDSEGLGISIIGMGAGADMGLEKLGIFVKTVTEGGAAHRDGRIQVN DLLVEVDGTSLVGVTQSFAASVLRNTKGRVRFMIGRER |
| PREX1_1 | NKQLRNDFKLVENILAKRLLILPQEEDYGFDIEEKNKAVVVKSVQRGSLAEVAGL QVGRKIYSINEDLVFLRPFSEVESILNQSFCSRRPLRLLVATKAKEIIKIPDQPDT |
| PREX1_2 | ATKAKEIIKIPDQPDTLCFQIRGAAPPYVYAVGRGSEAMAAGLCAGQCILKVNGSN VMNDGAPEVLEHFQAFRSRREEALGLYQ |
| PREX2_1 (DEPDC2) | HDLKVVENVIAKSLLIKSNEGSYGFGLEDKNKVPIIKLVEKGSNAEMAGMEVGKK IFAINGDLVFMRPFNEVDCFLKSCLNSRKPLRVLVSTKPRE |

| | |
|---|---|
| PREX2_2 | PLRVLVSTKPRETVKIPDSADGLGFQIRGFGPSVVHAVGRGTVAAAAGLHPGQCIIKVNGINVSKETHASVIAHVTACRKYRRPTKQDSIQWVYNSIESAQEDLQKSHSKP |
| PRX | MEARSRSAEELRRAELVEIIVETEAQTGVSGINVAGGGKEGIFVRELREDSPAARSLSLQEGDQLLSARVFFENFKYEDALRLLQCAEPYKVSFCLKRTVPTGDLALRPGTVSG |
| PSCDBP | RSSSLSDFSWSQRKLVTVEKQDNETFGFEIQSYRPQNQNACSSEMFTLICKIQEDSPAHCAGLQAGDVLANINGVSTEGFTYKQVVDLIRSSGNLLTIETLNGT |
| PSMD9 | KEKQARDMAEAHKEAMSRKLGQSESQGPPRAFAKVNSISPGSPASIAGLQVDDEIVEFGSVNTQNFQSLHNIGSVVQHSEGKPLNVTVIRRGEKHQLRLVPTRWAGKGLLGCNIIPLQR |
| PTPN13_1 | SSPEREITLVNLKKDAKYGLGFQIIGGEKMGRLDLGIFISSVAPGGPADLDGCLKPGDRLISVNSVSLEGVSHHAAIEILQNAPEDVTLVISQPKEKISKVPST |
| PTPN13_2 | KPGDIFEVELAKNDNSLGISVTVLFDKGGVNTSVRHGGIYVKAVIPQGAAESDGRIHKGDRVLAVNGVSLEGATHKQAVETLRNTGQVVHLLLEKGQSP |
| PTPN13_3 | TEENTFEVKLFKNSSGLGFSFSREDNLIPEQINASIVRVKKLFPGQPAAESGKIDVGDVILKVNGASLKGLSQQEVISALRGTAPEVFLLLCRPPPG |
| PTPN13_4 | EDFELEVELLITLIKSEKGSLGFTVTKGNQRIGCYVHDVIQDPAKSDGRLKPGDRLIKVNDTDVTNMTHTDAVNLLRAASKTVRLVIGRVLELPR |
| PTPN13_5 | PMLPHLLPDITLTCNKEELGFSLCGGHDSLYQVVYISDINPRSVAAIEGNLQLLDVIHYVNGVSTQGMTLEEVNRALDMSLPSLVLKATRNDLPVVPSS |
| PTPN3 | DSYLVLIRITPDEDGKFGFNLKGGVDQKMPLVVSRINPESPADTCIPKLNEGDQIVLINGRDISEHTHDQVVMFIKASRESHSRELALVIRRR |
| PTPN4 | DNLVLIRMKPDENGRFGFNVKGGYDQKMPVIVSRVAPGTPADLCVPRLNEGDQVVLINGRDIAEHTHDQVVLFIKASCERHSGELMLLVRP |
| RADIL | YVFTVELERGPSGLGMGLIDGMHTHLGAPGLYIQTLLPGSPAAADGRLSLGDRILEVNGSSLLGLGYLRAVDLIRHGGKKMRFLVAKSDVETAKKIHFRTPPL |
| RAPGEF2 | NIACAAKAKRRLMTLTKPSREAPLPFILLGGSEKGFGIFVDSVDSGSKATEAGLKRGDQILEVNGQNFENIQLSKAMEILRNNTHLSITVKTNLFVFKE |
| RAPGEF6 | KAKWRQVVLQKASRESPLQFSLNGGSEKGFGIFVEGVEPGSKAADSGLKRGDQIMEVNGQNFENITFMKAVEILRNNTHLALTVKTN |
| RGS12 | RAGEASKRPLPGPSPPRVRSVEVARGRAGYGFTLSGQAPCVLSCVMRGSPADFVGLRAGDQILAVNEINVKKASHEDVVKLIGKCSGVLHMVIAEGVGRFESCSSDEEGGLYEGKGWLK |
| RGS3 | SERRYRQITIPRGKDGFGFTICCDSPVRVQAVDSGGPAERAGLQQLDTVLQLNERPVEHWKCVELAHEIRSCPSEIILLVWRMVPQ |
| RHPN1 | AKNRWRLVGPVHLTRGEGGFGLTLRGDSPVLIAAVIPGSQAAAAGLKEGDYIVSVNGQPCRWWRHAEVVTELKAAGEAGASLQVVSLLPSSRLPSLGDR |
| RHPN2 | SANKRWTPPRSIRFTAEEGDLGFTLRGNAPVQVHFLDPYCSASVAGAREGDYIVSIQLVDCKWLTLSEVMKLLKSFGEDEIEMKVVSLLDSTSSMHNKSAT |
| RIMS1 | PVTWQPSKEGDRLIGRVILNKRTTMPKDSGALLGLKVVGGKMTDLGRLGAFITKVKKGSLADVVGHLRAGDEVLEWNGKPLPGATNEEVYNIILESKSEPQVEIIVSRP |

| | |
|---|---|
| RIMS2 | HSHSDKHPVTWQPSKDGDRLIGRILLNKRLKDGSVPRDSGAMLGLKVVGGKMTESGRLCAFITKVKKGSLADTVGHLRPGDEVLEWNGRLLQGATFEEVYNIILESKPEPQVELVVSRPIGD |
| SCRIB_1 | EPARIEEEELTLTILRQTGGLGISIAGGKGSTPYKGDDEGIFISRVSEEGPAARAGVRVGDKLLEVNGVALQGAEHHEAVEALRGAGTAVQMRVWRE |
| SCRIB_2 | RQRHVACLARSERGLGFSIAGGKGSTPYRAGDAGIFVSRIAEGGAAHRAGTLQVGDRVLSINGVDVTEARHDHAVSLLTAASPTIALLLERE |
| SCRIB_3 | PYPVEEIRLPRAGGPLGLSIVGGSDHSSHPFGVQEPGVFISKVLPRGLAARSGLRVGDRILAVNGQDVRDATHQEAVSALLRPCLELSLLVRRDP |
| SCRIB_4 | RRDPAPPGLRELCIQKAPGERLGISIRGGARGHAGNPRDPTDEGIFISKVSPTGAAGRDGRLRVGLRLLEVNQQSLLGLTHGEAVQLLRSVGDTLTVLVCDGFEASTD |
| SDCBP_1 | GIREVILCKDQDGKIGLRLKSIDNGIFVQLVQANSPASLVGLRFGDQVLQINGENCAGWSSDKAHKVLKQAFGEKITMTIRDRPFERT |
| SDCBP_2 | ERTITMHKDSTGHVGFIFKNGKITSIVKDSSAARNGLLTEHNICEINGQNVIGLKDSQIADILSTSGTVVTITIMPA |
| SDCBP2_1 | KPGVREIHLCKDERGKTGLRLRKVDQGLFVQLVQANTPASLVGLRFGDQLLQIDGRDCAGWSSHKAHQVVKKASGDKIVVVVRDRPFQRT |
| SDCBP2_2 | RPFQRTVTMHKDSMGHVGFVIKKGKIVSLVKGSSAARNGLLTNHYVCEVDGQNVIGLKDKKIMEILATAGNVVTLTIIPSVIYEH |
| SHANK1 | SDYIIKEKTVLLQKKDSEGFGFVLRGAKAQTPIEEFTPTPAFPALQYLESVDEGGVAWRAGLRMGDFLIEVNGQNVVKVGHRQVVNMIRQGGNTLMVKVVMVTRHPD |
| SHANK2 | SDCIIEEKTVVLQKKDNEGFGFVLRGAKADTPIEEFTPTPAFPALQYLESVDEGGVAWQAGLRTGDFLIEVNNENVVKVGHRQVVNMIRQGGNHLVLKVVTVTRNLDP |
| SHANK3 | HSDYVIDDKVAVLQKRDHEGFGFVLRGAKAETPIEEFTPTPAFPALQYLESVDVEGVAWRAGLRTGDFLIEVNGVNVVKVGHKQVVALIRQGGNRLVMKVVSVTRKPEE |
| SHROOM2 | DGGRLVEVQLSGGAPWGFTLKGGREHGEPLVITKIEEGSKAAAVDKLLAGDEIVGINDIGLSGFRQEAICLVKGSHKTLKLVVKRRSE |
| SHROOM3 | TKGRYIYLEAFLEGGAPWGFTLKGGLEHGEPLIISKVEEGGKADTLSSKLQAGDEVVHINEVTLSSSRKEAVSLVKGSYKTLRLVVRRDVCTD |
| SHROOM4 | SFQYVPVQLQGGAPWGFTLKGGLEHCEPLTVSKIEDGGKAALSQKMRTGDELVNINGTPLYGSRQEALILIKGSFRILKLIVRRRNAPV |
| SIPA1 | CETRELALPRDGQGRLGFEVDAEGFVTHVERFTFAETAGLRPGARLLRVCGQTLPSLRPEAAAQLLRSAPKVCVTVLPPDES |
| SIPA1L1 | SKGCESVEMTLRRNGLGQLGFHVNYEGIVADVEPYGYAWQAGLRQGSRLVEICKVAVATLSHEQMIDLLRTSVTVKVVIIPPHDD |
| SIPA1L2 | TRGCETVEMTLRRNGLGQLGFHVNFEGIVADVEPFGFAWKAGLRQGSRLVEICKVAVATLTHEQMIDLLRTSVTVKVVIIQPHDD |
| SIPA1L3 | TSGWETVDMTLRRNGLGQLGFHVKYDGTVAEVEDYGFAWQAGLRQGSRLVEICKVAVVTLTHDQMIDLLRTSVTVKVVIIPPFED |
| SNTA1 | QRRRVTVRKADAGGLGISIKGGRENKMPILISKIFKGLAADQTEALFVGDAILSVNGEDLSSATHDEAVQVLKKTGKEVVLEVKYMKD |

| | |
|---|---|
| SNTB1 | SNQKRGVKVLKQELGGLGISIKGGKENKMPILISKIFKGLAADQTQALYVGDAILSVNGADLRDATHDEAVQALKRAGKEVLLEVKYMRE |
| SNTB2 | PVRRVRVVKQEAGGLGISIKGGRENRMPILISKIFPGLAADQSRALRLGDAILSVNGTDLRQATHDQAVQALKRAGKEVLLEVKFIRE |
| SNTG1 | GERTVTIRRQTVGGFGLSIKGGAEHNIPVVVSKISKEQRAELSGLLFIGDAILQINGINVRKCRHEEVVQVLRNAGEEVTLTVSFLKRAP |
| SNTG2 | NRRTVTLRRQPVGGLGLSIKGGSEHNVPVVISKIFEDQAADQTGMLFVGDAVLQVNGIHVENATHEEVVHLLRNAGDEVTITVEYLREAP |
| SNX27 | GPRVVRIVKSESGYGFNVRGQVSEGGQLRSINGELYAPLQHVSAVLPGGAADRAGVRKGDRILEVNHVNVEGATHKQVVDLIRAGEKELILTVLSVPPHEAD |
| STXB4 | EKDPAFQMITIAKETGLGLKVLGGINRNEGPLVYIQEIIPGGDCYKDGRLKPGDQLVSVNKESMIGVSFEEAKSIITGAKLRLESAWEIAFIRQKSDN |
| SYNJ2BP | DYLVTEEEINLTRGPSGLGFNIVGGTDQQYVSNDSGIYVSRIKENGAAALDGRLQEGDKILSVNGQDLKNLLHQDAVDLFRNAGYAVSLRVQHRLQVQ |
| SYNP2 | GTGDFICISMTGGAPWGFRLQGGKEQKQPLQVAKIRNQSKASGSGLCEGDEVVSINGNPCADLTYPEVIKLMESITDSLQMLIKRPSSG |
| SYNPO2L | MGAEEEVLVTLSGGAPWGFRLHGGAEQRKPLQVSKIRRRSQAGRAGLRERDQLLAINGVSCTNLSHASAMSLIDASGNQLVLTVQRLAD |
| TIAM1 | EIEICPKVTQSIHIEKSDTAADTYGFSLSSVEEDGIRRLYVNSVKETGLASKKGLKAGDEILEINNRAADALNSSMLKDFLSQPSLGLLVRTYPELEEGVE |
| TIAM2 | YDEIEVFPLNVYDVQLTKTGSVCDFGFAVTAQVDERQHLSRIFISDVLPDGLAYGEGLRKGNEIMTLNGEAVSDLDLKQMEALFSEKSVGLTLIARPPDTKAT |
| TJP1_1 | EETAIWEQHTVTLHRAPGFGFGIAISGGRDNPHFQSGETSIVISDVLKGGPAEGQLQENDRVAMVNGVSMDNVEHAFAVQQLRKSGKNAKITIRRKKKVQ |
| TJP1_2 | PTKVTLVKSRKNEEYGLRLASHIFVKEISQDSLAARDGNIQEGDVVLKINGTVTENMSLTDAKTLIERSKGKLKMVVQRDE |
| TJP1_3 | DGILRPSMKLVKFRKGDSVGLRLAGGNDVGIFVAGVLEDSPAAKEGLEEGDQILRVNNVDFTNIIREEAVLFLLDLPKGEEVTILAQKKKDVYRRIVESDVG |
| TJP2_1 | MEELIWEQYTVTLQKDSKRGFGIAVSGGRDNPHFENGETSIVISDVLPGGPADGLLQENDRVVMVNGTPMEDVLHSFAVQQLRKSGKVAAIVVKRPRKVQVAALQ |
| TJP2_2 | RGRPGPIGVLLMKSRANEEYGLRLGSQIFVKEMTRTGLATKDGNLHEGDIILKINGTVTENMSLTDARKLIEKSRGKLQLVVLRDSQQT |
| TJP2_3 | EDEAIYGPNTKMVRFKKGDSVGLRLAGGNDVGIFVAGIQEGTSAEQEGLQEGDQILKVNTQDFRGLVREDAVLYLLEIPKGEMVTILAQSRADVYRD |
| TJP3_1 | MEELTIWEQHTATLSKDPRRGFGIAISGGRDRPGGSMVVSDVVPGGPAEGRLQTGDHIVMVNGVSMENATSAFAIQILKTCTKMANITVKRPRRIHLPATKASPSSPGR |
| TJP3_2 | QMKPVKSVLVKRRDSEEFGVKLGSQIFIKHITDSGLAARHRGLQEGDLILQINGVSSQNLSLNDTRRLIEKSEGKLSLLVLRDRGQ |
| TJP3_3 | EDRGYSPDTRVVRFLKGKSIGLRLAGGNDVGIFVSGVQAGSPADGQGIQEGDQILQVNDVPFQNLTREEAVQFLLGLPPGEEMELVTQRKQDIFWKMVQSRVGDSFYIRT |
| TX1B3 | QPVTAVVQRVEIHKLRQGENLILGFSIGGGIDQDPSQNPFSEDKTDKGIYVTRVSEGGPAEIAGLQIGDKIMQVNGWDMTMVTHDQARKRLTKRSEEVVRLLVTRQSLQKAVQQSMLS |

| | |
|---|---|
| USH1C_1 | DQLTPRRSRKLKEVRLDRLHPEGLGLSVRGGLEFGCGLFISHLIKGGQADSVGLQVGDEIVRINGYSISSCTHEEVINLIRTKKTVSIKVRHIGLIPVKSSPDE |
| USH1C_2 | KEKKVFISLVGSRGLGCSISSGPIQKPGIFISHVKPGSLSAEVGLEIGDQIVEVNGVDFSNLDHKEAVNVLKSSRSLTISIVAAAGRELFMT |
| USH1C_3 | SMFTPEQIMGKDVRLLRIKKEGSLDLALEGGVDSPIGKVVVSAVYERGAAERHGGIVKGDEIMAINGKIVTDYTLAEAEAALQKAWNQGGDWIDLVVAVCPPKEYDDE |

Individual single PDZ domains used as prey in the Y2H screens. Note that the clones presented with another name in the previous version of the PDZome are indicated in gray (in parentheses) below the name used in the present version.

**Table S2 PDZ constructs used as preys to comprehensively map PDZ interactions.**

| PDZ tandem name | Protein Sequence |
|---|---|
| APBA1_1-2 | EFKDVFIEKQKGEILGVVIVESGWGSILPTVIIANMMHGGPAEKSGKLNIGDQIMSINGT<br>SLVGLPLSTCQSIIKGLKNQSRVKLNIVRCPPVTTVLIRRPDLRYQLGFSVQNGIICSLMR<br>GGIAERGGVRVGHRIIEINGQSVVATPHEKIVHILSNAVGEIHMKTMPA |
| APBA2_1-2 | NCKELQLEKHKGEILGVVVVESGWGSILPTVILANMMNGGPAARSGKLSIGDQIMSIN<br>GTSLVGLPLATCQGIIKGLKNQTQVKLNIVSCPPVTTVLIKRPDLKYQLGFSVQNGIICS<br>LMRGGIAERGGVRVGHRIIEINGQSVVATAHEKIVQALSNSVGEIHMKTMPAA |
| APBA3_1-2 | DNCREVHLEKRRGEGLGVALVESGWGSLLPTAVIANLLHGGPAERSGALSIGDRLTAI<br>NGTSLVGLPLAACQAAVRETKSQTSVTLSIVHCPVTTAIIHRPHAREQLGFCVEDGIICS<br>LLRGGIAERGGIRVGHRIIEINGQSVVATPHARIIELLTEAYGEVHIKTMPAATYRLLTG<br>Q |
| DFNB31_1-2 | GEVRLVSLRRAKAHEGLGFSIRGGSEHGVGIYVSLVEPGSLAEKEGLRVGDQILRVND<br>KSLARVTHAEAVKALKGSKKLVLSVYSAGRIPGGYVTNHIYTWVDPQGRSISPPSGLP<br>QPHGGALRQQEGDRRSTLHLLQGGDEKKVNLVLGDGRSLGLTIRGGAEYGLGIYITGV<br>DPGSEAEGSGLKVGDQILEVNGRSFLNILHDEAVRLLKSSRHLILTVKDVGRLPRARTT<br>VDETKWIASSR |
| DLG1_1-2 | EYEEITLERGNSGLGFSIAGGTDNPHIGDDSSIFITKIITGGAAAQDGRLRVNDCILRVNE<br>VDVRDVTHSKAVEALKEAGSIVRLYVKRRKPVSEKIMEIKLIKGPKGLGFSIAGGVGN<br>QHIPGDNSIYVTKIIEGGAAHKDGKLQIGDKLLAVNNVCLEEVTHEEAVTALKNTSDF<br>VYLKVAKPTSMYMND |
| DLG2_1-2 | EYEFEEITLERGNSGLGFSIAGGTDNPHIGDDPGIFITKIIPGGAAAEDGRLRVNDCILRV<br>NEVDVSEVSHSKAVEALKEAGSIVRLYVRRRRPILETVVEIKLFKGPKGLGFSIAGGVG<br>NQHIPGDNSIYVTKIIDGGAAQKDGRLQVGDRLLMVNNYSLEEVTHEEAVAILKNTSE<br>VVYLKVGKPTTIY |
| DLG3_1-2 | DGMFKYEEIVLERGNSGLGFSIAGGIDNPHVPDDPGIFITKIIPGGAAAMDGRLGVNDC<br>VLRVNEVDVSEVVHSRAVEALKEAGPVVRLVVRRRQPPPETIMEVNLLKGPKGLGFSI<br>AGGIGNQHIPGDNSIYITKIIEGGAAQKDGRLQIGDRLLAVNNTNLQDVRHEEAVASLK<br>NTSDMVYLKVAKPGS |
| DLG4_1-2 | EYEEITLERGNSGLGFSIAGGTDNPHIGDDPSIFITKIIPGGAAAQDGRLRVNDSILFVNE<br>VDVREVTHSAAVEALKEAGSIVRLYVMRRKPPAEKVMEIKLIKGPKGLGFSIAGGVGN<br>QHIPGDNSIYVTKIIEGGAAHKDGRLQIGDKILAVNSVGLEDVMHEDAVAALKNTYDV<br>VYLKVAKPSNA |
| DLG5_1-2 | ETEVVEFERETEDIDLKALGFDMAEGVNEPCFPGDCGIFVTKVDKGSIADGRLRVNDW<br>LLRINDVDLINKDKKQAIKALLNGEGAINMVVRRRKSLGGKVVTPLHINLSGQKDSGIS<br>LENGVYAAAVLPGSPAAKEGSLAVGDRIVAINGIALDNKSLNECESLLRSCQDSLTLSL<br>LKVFPQSS |
| GRIP1_1-2 | EFKGSTVVELMKKEGTTLGLTVSGGIDKDGKPRVSNLRQGGIAARSDQLDVGDYIKA<br>VNGINLAKFRHDEIISLLKNVGERVVLEVEYELPPVSVQGSSVIFRTVEVTLHKEGNTFG<br>FVIRGGAHDDRNKSRPVVITCVRPGGPADREGTIKPGDRLLSVDGIRLLGTTHAEAMSI<br>LKQCGQEAALLIEYDVS |
| GRIP1_2-3 | SVIFRTVEVTLHKEGNTFGFVIRGGAHDDRNKSRPVVITCVRPGGPADREGTIKPGDRL<br>LSVDGIRLLGTTHAEAMSILKQCGQEAALLIEYDVSVMDSVATASGPLLVEVAKTPGA<br>SLGVALTTSMCCNKQVIVIDKIKSASIADRCGALHVGDHILSIDGTSMEYCTLAEATQF<br>LANTTDQVKLEILPHHQTRLALKG |
| GRIP1_4-5 | QVVHTETTEVVLTADPVTGFGIQLQGSVFATETLSSPPLISYIEADSPAERCGVLQIGDR<br>VMAINGIPTEDSTFEEASQLLRDSSITSKVTLEIEFDVAESVIPSSGTFHVKLPKKHNVEL<br>GITISSPSSRKPGDPLVISDIKKGSVAHRTGTLELGDKLLAIDNIRLDNCSMEDAVQILQQ<br>CEDLVKLKIRKDED |
| GRIP1_5-6 | ESVIPSSGTFHVKLPKKHNVELGITISSPSSRKPGDPLVISDIKKGSVAHRTGTLELGDKL<br>LAIDNIRLDNCSMEDAVQILQQCEDLVKLKIRKDEDNSDEQESSGAIIYTVELKRYGGP<br>LGITISGTEEPFDPIIISSLTKGGLAERTGAIHIGDRILAINSSSLKGKPLSEAIHLLQMAGE<br>TVTLKIKKQTDAQSASS |
| GRIP2_1-2 | EEFRGITVVELIKKEGSTLGLTISGGTDKDGKPRVSNLRPGGLAARSDLLNIGDYIRSVN<br>GIHLTRLRHDEIITLLKNVGERVVLEVEYELPPPAPENNPRIISKTVDVSLYKEGNSFGFV |

| | |
|---|---|
| | LRGGAHEDGHKSRPLVLTYVRPGGPADREGSLKVGDRLLSVDGIPLHGASHATALATL RQCSHEALFQVEYDVATPDTVANASG |
| GRIP2_2-3 | ENNPRIISKTVDVSLYKEGNSFGFVLRGGAHEDGHKSRPLVLTYVRPGGPADREGSLK VGDRLLSVDGIPLHGASHATALATLRQCSHEALFQVEYDVATPDTVANASGPLMVEIV KTPGSALGISLTTTSLRNKSVITIDRIKPASVVDRSGALHPGDHILSIDGTSMEHCSLLEA TKLLASISEKVRLEILPVPQSQRPLR |
| GRIP2_4-5 | GGQIVHTETTEVVLCGDPLSGFGLQLQGGIFATETLSSPPLVCFIEPDSPAERCGLLQVG DRVLSINGIATEDGTMEEANQLLRDAALAHKVVLEVEFDVAESVIPSSGTFHVKLPKK RSVELGITISSASRKRGEPLIISDIKKGSVAHRTGTLEPGDKLLAIDNIRLDNCPMEDAVQ ILRQCEDLVKLKIRKDED |
| GRIP2_5-6 | DVAESVIPSSGTFHVKLPKKRSVELGITISSASRKRGEPLIISDIKKGSVAHRTGTLEPGD KLLAIDNIRLDNCPMEDAVQILRQCEDLVKLKIRKDEDNSDELETTGAVSYTVELKRY GGPLGITISGTEEPFDPIVISGLTKRGLAERTGAIHVGDRILAINNVSLKGRPLSEAIHLLQ VAGETVTLKIKKQLDR |
| INADL_1-2 | KLGNEDFNSVIQQMAQGRQIEYIDIERPSTGGLGFSVVALRSQNLGKVDIFVKDVQPGS VADRDQRLKENDQILAINHTPLDQNISHQQAIALLQQTTGSLRLIVAREPVHTKSSTSSS LNDTTLPETVCWGHVEEVELINDGSGLGFGIVGGKTSGVVVRTIVPGGLADRDGRLQT GDHILKIGGTNVQGMTSEQVAQVLRNCGNSVRMLVARDPAGDISVT |
| INADL_2-3 | PETVCWGHVEEVELINDGSGLGFGIVGGKTSGVVVRTIVPGGLADRDGRLQTGDHILK IGGTNVQGMTSEQVAQVLRNCGNSVRMLVARDPAGDISVTPPAPAALPVALPTVASK GPGSDSSLFETYNVELVRKDGQSLGIRIVGYVGTSHTGEASGIYVKSIIPGSAAYHNGHI QVNDKIVAVDGVNIQGFANHDVVEVLRNAGQVVHLTLVRRKTSSSTSPLEPPSDRGT |
| INADL_4-5 | DTQIADDAELQKYSKLLPIHTLRLGVEVDSFDGHHYISSIVSGGPVDTLGLLQPEDELLE VNGMQLYGKSRREAVSFLKEVPPPFTLVCCRRLFDDEASVDEPRRTETSLPETEVDHN MDVNTEEDDDGELALWSPEVKIVELVKDCKGLGFSILDYQDPLDPTRSVIVIRSLVAD GVAERSGGLLPGDRLVSVNEYCLDNTSLAEAVEILKAVPPGLVHLGICKPLVEDNEEE |
| INADL_8-9 | PATCPIVPGQEMIIEISKGRSGLGLSIVGGKDTPLNAIVIHEVYEEGAAARDGRLWAGD QILEVNGVDLRNSSHEEAITALRQTPQKVRLVVYRDEAHYRDEENLEIFPVDLQKKAG RGLGLSIVGKRNGSGVFISDIVKGGAADLDGRLIQGDQILSVNGEDMRNASQETVATIL KCAQGLVQLEIGRLR |
| LNX1_1-2 | PRLYHLIPDGEITSIKINRVDPSESLSIRLVGGSETPLVHIIIQHIYRDGVIARDGRLLPGDII LKVNGMDISNVPHNYAVRLLRQPCQVLWLTVMREQKFRSRNNGQAPDAYRPRDDSF HVILNKSSPEEQLGIKLVRKVDEPGVFIFNVLDGGVAYRHGQLEENDRVLAINGHDLR YGSPESAAHLIQASERRVHLVVSRQVRQRSPD |
| LNX2_1-2 | PLSLPEGEITTIEIHRSNPYIQLGISIVGGNETPLINIVIQEVYRDGVIARDGRLLAGDQILQ VNNYNISNVSHNYARAVLSQPCNTLHLTVLRERRFGNRAHNHSDSNSPREEIFQVALH KRDSGEQLGIKLVRRTDEPGVFILDLLEGGLAAQDGRLSSNDRVLAINGHDLKYGTPE LAAQIIQASGERVNLTIARPGKPQP |
| MPDZ_1-2 | DEFDQLIKNMAQGRHVEVFELLKPPSGGLGFSVVGLRSENRGELGIFVQEIQEGSVAHR DGRLKETDQILAINGQALDQTITHQQAISILQKAKDTVQLVIARGSLPQLVSPIVSRSPSA ASTISAHSNPVHWQHMETIELVNDGSGLGFGIIGGKATGVIVKTILPGGVADQHGRLCS GDHILKIGDTDLAGMSSEQVAQVLRQCGNRVKLMIARGAIEERT |
| MPDZ_8-9 | DKEDEFGYSWKNIRERYGTLTGELHMIELEKGHSGLGLSLAGNKDRSRMSVFIVGIDP NGAAGKDGRLQIADELLEINGQILYGRSHQNASSIIKCAPSKVKIIFIRNKDAVNQMAV CPGNAVEPLPSNSENLQNKETEPTVTTSDAAVDLSSFKNVQHLELPKDQGGLGIAISEE DTLSGVIIKSLTEHGVAATDGRLKVGDQILAVDDEIVVGYPIEKFISLLKTAKMTVKLTI HAENPDSQAVPS |
| MPDZ_10-11 | PGCETTIEISKGRTGLGLSIVGGSDTLLGAIIIHEVYEEGAACKDGRLWAGDQILEVNGI DLRKATHDEAINVLRQTPQRVRLTLYRDEAPYKEEEVCDTLTIELQKKPGKGLGLSIVG KRNDTGVFVSDIVKGGIADADGRLMQGDQILMVNGEDVRNATQEAVAALLKCSLGT VTLEVGRIKAGP |
| NHERF3_2-3 | QPRLCYLVKEGGSYGFSLKTVQGKKGVYMTDITPQGVAMRAGVLADDHLIEVNGEN VEDASHEEVVEKVKKSGSRVMFLLVDKETDKRHVEQKIQFKRETASLKLLPHQPRIVE MKKGSNGYGFYLRAGSEQKGQIIKDIDSGSPAEEAGLKNNDLVVAVNGESVETLDHD SVVEMIRKGGDQTSLLVVDKETDNMYR |
| NHERF4_1-2 | DPYDPWSLERPRFCLLSKEEGKSFGFHLQQELGRAGHVVCRVDPGTSAQRQGLQEGD RILAVNNDVVEHEDYAVVVRRIRASSPRVLLTVLARHAHDVARAQLGEDAHLCPTLG PGVRPRLCHIVKDEGGFGFSVTHGNQGPFWLVLSTGGAAERAGVPPGARLLEVNGVS VEKFTHNQLTRKLWQSGQQVTLLVAGPEVEEQCR |

| | |
|---|---|
| PARD3B_2-3 | NKNAKKIKIDLKKGPEGLGFTVVTRDSSIHGPGPIFVKNILPKGAAIKDGRLQSGDRILE VNGRDVTGRTQEELVAMLRSTKQGETASLVIARQEGHFLPRELKGEPDCCALSLETSE QLTFEIPLNDSGSAGLGVSLKGNKSRETGTDLGIFIKSIIHGGAAFKDGRLRMNDQLIAV NGESLLGKSNHEAMETLRRSMSMEGNIRGMIQLVILRRPER |
| PDZD2_5-6 | KAQSENEEDVCFIVLNRKEGSGLGFSVAGGTDVEPKSITVHRVFSQGAASQEGTMNRG DFLLSVNGASLAGLAHGNVLKVLHQAQLHKDALVVIKKGMDQPRPSARQEPPTANG KGLLSRKTIPLEPGIGRSVAVHDALCVEVLKTSAGLGLSLDGGKSSVTGDGPLVIKRVY KGGAAEQAGIIEAGDEILAINGKPLVGLMHFDAWNIMKSVPEGPVQLLIRKHRNSS |
| PDZD7_1-2 | DIIHSVRVEKSPAGRLGFSVRGGSEHGLGIFVSKVEEGSSAERAGLCVGDKITEVNGLSL ESTTMGSAVKVLTSSSRLHMMVRRMGRVPGIKFSKEKTTWVDVVNRRLVVEKCGSTP SDTSSEDGVRRIVHLYTTSDDFCLGFNIRGGKEFGLGIYVSKVDHGGLAEENGIKVGDQ VLAANGVRFDDISHSQAVEVLKGQTHIMLTIKETGRYPAYKEMVSEYCWLDRLSNG |
| PREX1_1-2 | NKQLRNDFKLVENILAKRLLILPQEEDYGFDIEEKNKAVVVKSVQRGSLAEVAGLQVG RKIYSINEDLVFLRPFSEVESILNQSFCSRRPLRLLVATKAKEIIKIPDQPDTLCFQIRGAA PPYVYAVGRGSEAMAAGLCAGQCILKVNGSNVMNDGAPEVLEHFQAFRSRREEALGL YQ |
| PREX2_1-2 | HDLKVVENVIAKSLLIKSNEGSYGFGLEDKNKVPIIKLVEKGSNAEMAGMEVGKKIFAI NGDLVFMRPFNEVDCFLKSCLNSRKPLRVLVSTKPRETVKIPDSADGLGFQIRGFGPSV VHAVGRGTVAAAAGLHPGQCIIKVNGINVSKETHASVIAHVTACRKYRRPTKQDSIQW VYNSIESAQEDLQKSHSKP |
| PTPN13_4-5 | EDFELEVELLITLIKSEKGSLGFTVTKGNQRIGCYVHDVIQDPAKSDGRLKPGDRLIKVN DTDVTNMTHTDAVNLLRAASKTVRLVIGRVLELPRIPMLPHLLPDITLTCNKEELGFSL CGGHDSLYQVVYISDINPRSVAAIEGNLQLLDVIHYVNGVSTQGMTLEEVNRALDMSL PSLVLKATRNDLPVVPSS |
| SCRIB_3-4 | LAAALEGPYPVEEIRLPRAGGPLGLSIVGGSDHSSHPFGVQEPGVFISKVLPRGLAARSG LRVGDRILAVNGQDVRDATHQEAVSALLRPCLELSLLVRRDPAPPGLRELCIQKAPGE RLGISIRGGARGHAGNPRDPTDEGIFISKVSPTGAAGRDGRLRVGLRLLEVNQQSLLGL THGEAVQLLRSVGDTLTVLVCDGFEASTDAALEVS |
| SCRIB_3-4_L | PYPVEEIRLPRAGGPLGLSIVGGSDHSSHPFGVQEPGVFISKVLPRGLAARSGLRVGDRI LAVNGQDVRDATHQEAVSALLRPCLELSLLVRRDPAPPGLRELCIQKAPGERLGISIRG GARGHAGNPRDPTDEGIFISKVSPTGAAGRDGRLRVGLRLLEVNQQSLLGLTHGEAVQ LLRSVGDTLTVLVCDGFEASTD |
| USH1C_1-2 | TPRRSRKLKEVRLDRLHPEGLGLSVRGGLEFGCGLFISHLIKGGQADSVGLQVGDEIVR INGYSISSCTHEEVINLIRTKKTVSIKVRHIGLIPVKSSPDEPLTWQYVDQFVSESGGVRG SLGSPGNRENKEKKVFISLVGSRGLGCSISSGPIQKPGIFISHVKPGSLSAEVGLEIGDQIV EVNGVDFSNLDHKEAVNVLKSSRSLTISIVAAAGRELFMT |
| SDCBP_1-2 § | GIREVILCKDQDGKIGLRLKSIDNGIFVQLVQANSPASLVGLRFGDQVLQINGENCAGW SSDKAHKVLKQAFGEKITMTIRDRPFERTERTITMHKDSTGHVGFIFKNGKITSIVKDSS AARNGLLTEHNICEINGQNVIGLKDSQIADILSTSGTVVTITIMPA |
| SDCBP2_1-2 § | KPGVREIHLCKDERGKTGLRLRKVDQGLFVQLVQANTPASLVGLRFGDQLLQIDGRD CAGWSSHKAHQVVKKASGDKIVVVVRDRPFQRTRPFQRTVTMHKDSMGHVGFVIKK GKIVSLVKGSSAARNGLLTNHYVCEVDGQNVIGLKDKKIMEILATAGNVVTLTIIPSVI YEH |

Tandem PDZ domains used as prey in the Y2H screens. Note that the tandems for Syntenin-1 and 2 are not present in Duhoo et al 2020 (§).
